# High Species Richness of *Scinax* Treefrogs (Hylidae) in a Threatened Amazonian Landscape Revealed by an Integrative Approach

**DOI:** 10.1371/journal.pone.0165679

**Published:** 2016-11-02

**Authors:** Miquéias Ferrão, Olavo Colatreli, Rafael de Fraga, Igor L. Kaefer, Jiří Moravec, Albertina P. Lima

**Affiliations:** 1 Programa de Pós-Graduação em Ecologia, Instituto Nacional de Pesquisas da Amazônia, Manaus, Amazonas, Brazil; 2 Coordenação de Biodiversidade, Instituto Nacional de Pesquisas da Amazônia, Manaus, Amazonas, Brazil; 3 Instituto de Ciências Biológicas, Universidade Federal do Amazonas, Manaus, Amazonas, Brazil; 4 Department of Zoology, National Museum, Prague, Czech Republic; University of Arkansas, UNITED STATES

## Abstract

Rising habitat loss is one of the main drivers of the global amphibian decline. Nevertheless, knowledge of amphibian diversity needed for effective habitat protection is still highly inadequate in remote tropical regions, the greater part of the Amazonia. In this study we integrated molecular, morphological and bioacoustic evidence to evaluate the species richness of the treefrogs genus *Scinax* over a 1000 km transect across rainforest of the Purus-Madeira interfluve, and along the east bank of the upper Madeira river, Brazilian Amazonia. Analysis revealed that 82% of the regional species richness of *Scinax* is still undescribed; two nominal species, seven confirmed candidate species, two unconfirmed candidate species, and one deep conspecific lineage were detected in the study area. DNA barcoding based analysis of the 16s rRNA gene indicates possible existence of three discrete species groups within the genus *Scinax*, in addition to the already-known *S*. *rostratus* species Group. Quantifying and characterizing the number of undescribed *Scinax* taxa on a regional scale, we provide a framework for future taxonomic study in Amazonia. These findings indicate that the level to which Amazonian anura species richness has been underestimated is far greater than expected. Consequently, special attention should be paid both to taxonomic studies and protection of the still-neglected Amazonian *Scinax* treefrogs.

## Introduction

Frogs achieve mega-diversity in the tropics, but this extreme species richness is under strong pressure from human disturbance, mainly via habitat loss and degradation of breeding sites [1]. Contemporaneously, it is becoming increasingly evident that the diversity of frogs has been severely underestimated, and that this is true in many different anuran groups. For instance, the species richness in the genus *Adenomera* Steindachner, 1867, in the subfamily *Phyzelaphryninae* Hedges, Duellman and Heinicke, 2008 and in the genus *Osteocephalus* Steindachner, 1862 increased by 116%, 100% and 37.5%, respectively [2–4]. Moreover, species richness in the genus *Engystomops* Jimenez de la Espada, 1872 and in the *Hypsiboas calcaratus*–*Hypsiboas fasciatus* species complex increased by at least 150% and 200%, respectively [5–6] (see S1 Appendix for cryptic diversity estimates). Finally, 11 distinct lineages of leaf-frogs of the *Rhinella margaritifera* species complex and six lineages of treefrogs of the *Scinax ruber* species complex have been identified as potentially new species [7].

Taxonomic studies of the genus *Scinax* Wagler, 1830 are very challenging due to the large number of morphologically similar species, especially those belonging to the same species complexes [8–10]. In addition to difficulties in species recognition, the high number of cryptic species [7] and the lack of information about the geographical range of many species, call for the introduction of non-morphological methods into research on the taxonomy of the genus. Such integrative taxonomic studies of Amazonian frogs have usually addressed morphological, molecular, bioacoustic and natural history data (e.g. [11–13, 6, 14–15]). The integration of different lines of evidence is a powerful tool for solving taxonomic problems (e.g. [10, 11, 13]) and understanding evolutionary relationships between taxa [16].

At present, the genus *Scinax* comprises 63 species of small and medium-sized arboreal frog (15–52 mm), and is distributed throughout the Americas from Mexico to Argentina [17]. Until 2014, 29 species of the *Scinax ruber* Clade (*sensu* [18]) were known from Amazonia (see [19]). However, *S*. *parkeri* (Gaige, 1929) and *S*. *trilineatus* (Hoogmoed and Gorzula, 1977) were recently synonymized with *S*. *fuscomarginatus* (Lutz, 1925), *S*. *madeirae* (Bokermann, 1964) was revalidated and *S*. *villasboasi* Brusquetti, Jansen, Barrio-Amarrós, Segalla and Haddad, 2014 was described from the eastern Brazilian Amazonia [10]. Therefore, 28 valid species of *Scinax* currently occur in Amazonia. Of these, seven species are placed in the *S*. *rostratus* species Group: *S*. *garbei* (Miranda-Ribeiro, 1926), *S*. *jolyi* Lescure and Marty, 2000, *S*. *kennedyi* (Pyburn, 1973), *S*. *nebulosus* (Spix, 1824), *S*. *pedromedinae* (Henle, 1991), *S*. *proboscideus* (Brongersma, 1933) and *S*. *rostratus* (Peters, 1863). Twenty-one species are not associated with any known species group (*sensu* [18]): *S*. *baumgardneri* (Rivero, 1961), *S*. *blairi* (Fouquette and Pyburn, 1972), *S*. *boesemani* (Goin, 1966), *S*. *chiquitanus* (De la Riva, 1990), *S*. *cruentommus* (Duellman, 1972), *S*. *danae* (Duellman, 1986), *S*. *exiguus* (Duellman, 1986), *S*. *funereus* (Cope, 1874), *S*. *fuscomarginatus*, *S*. *fuscovarius* (A. Lutz, 1925), *S*. *ictericus* Duellman and Wiens, 1993, *S*. *iquitorum* Moravec, Tuanama, Pérez and Lehr, 2009, *S*. *karenanneae* (Pyburn, 1992), *S*. *lindsayi* Pyburn, 1992, *S*. *madeirae*, *S*. *oreites* Duellman and Wiens, 1993, *S*. *ruber* (Laurenti, 1768), *S*. *sateremawe* Sturaro and Peloso, 2014, *S*. *villasboasi*, *S*. *wandae* (Pyburn and Fouquette, 1971) and *S*. *x-signatus* (Spix, 1824).

The number of currently-known species in the *S*. *ruber* Clade means it is already relatively species-rich. However, because evolutionary relationships among the species are still poorly known, addtional undescribed species may occur in this clade [7, 10, 20–21]. Our herpetological survey in southern Amazonia indicated that *Scinax* species richness has been underestimated, especially in poorly-sampled regions of the Purus-Madeira interfluve, and the east bank of the upper Madeira river, which are currently threatened by an extensive deforestation [22–24]. In this study, we investigated the species richness of *Scinax* treefrogs in the Purus-Madeira interfluve and along the eastern bank of the upper Madeira river, combining molecular, morphological and bioacoustic evidence.

## Materials and Methods

### Study area

**Fig 1:**
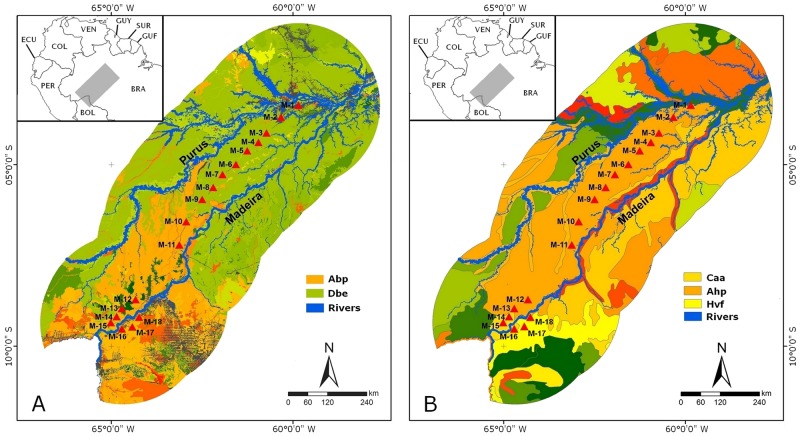
Sampling area in the Purus-Madeira interfluve and on the east bank of the upper Madeira river, Brazilian Amazonia. (A) Vegetation cover types. (B) Soil types. Abbreviations: BR, Brazil; M1–M18, RAPELD sampling modules; Abp, lowland ombrophilous open forest with palm trees; Dbe, lowland ombrophilous dense forest with emergent canopy; Caa, Chromic-Alumic Acrisol; Ahp, Alumic-Humic Plinthosol; Hvf, Hyperdystri-Vetic Ferralsol. The small insert in each map shows part of South America and abbreviated names of countries. The transverse gray bar represents the study area and adjacent territory.

The study area is located south of the Amazon river in the Brazilian states of Amazonas and Rondônia, and includes the interfluve between the Purus and Madeira rivers (PMIR) and the eastern bank of the upper Madeira (EBMR; Fig 1). It covers an area of approximately 15.4 million hectares and contains a complex network of water bodies [24]. The soil is mostly characterized as plinthosols [25], with a predominance of silt [26]. The Madeira river is the main tributary of the Amazon and its basin covers approximately 1.4 million km^2^. On a regional scale, the topography of PMIR and RBMD is relatively flat, with elevation ranging from 27 to 80 m amsl. At the local scale, elevation ranges between one and three meters, promoting the occurrence of temporary ponds in lower-lying areas during the rainy season [27]. On a coarse scale, the northern portion of PMIR is covered by tropical lowland rainforest with emergent canopy, while open lowland rainforest with palm trees occurs in the southern portion of PMIR and EBMR ([28], Fig 1A).

### Sampling design, collection effort and ethics

We collected data in 18 RAPELD sampling modules [29] distributed across a geographical transect of approximately 1000 km transect (15 modules in the PMIR and three modules in the EBMR; Fig 1). Each sampling module (hereafter abbreviated as M, followed by the number of each sampling unit) contained two parallel trails of 5 km each, spaced 1 km apart. Each trail contained five plots with a standardized area of 2500 m^2^ (250 m in length and 10 m in wide), totaling 10 plots per module. To minimize environmental heterogeneity within each sampling unit, the outline of each plot followed local topographic contours [29].

We sampled frogs using visual search (adapted from [30]) and auditory searches for calling males. Plots from M1–M11 were sampled three times each during the rainy season (November–March) between 2013 and 2014, and the plots M12–M18 were sampled three times each during the rainy season (October–March) between 2011 and 2014. In addition, we collected adults and tadpoles when this were encountered on the individual trails and the areas surrounding the sampling modules. Adults and tadpoles were anesthetized and killed with a solution of 10% benzocaine. After death, adults were fixed in 10% formaldehyde solution and stored in 70% ethanol, tadpoles were fixed and stored in 5% formaldehyde solution. Specimens were deposited in the herpetological collection of Instituto Nacional de Pesquisas da Amazônia (INPA-H), Manaus, Amazonas, Brazil (S2 Appendix).

Specimens were collected from M12 to M18 under IBAMA/SISBIO permit number 02001.000508/ 2008–99, and from M1 to M11 under ICMBio/RAN permit number Reg. 659755 Number 13777. IBAMA and ICMBio are institutes of Ministry of Environment, Government of Brazil. These permits were subject to approval of all procedures for collecting and euthanizing frogs.

### DNA extraction and sequence alignment

Genomic DNA was extracted from muscle tissue using the Wizard Genomic DNA Purification Kit (Promega Corporation, USA), following the manufacturer's protocol. We used the 16sa-L (or 16sar-L) (CGC CTG TTT ATC AAA AAC AT) and the 16sbr-H (GCC GTC TGA ACT CAG ATC GCA T) primers [31] to amplify ribosomal DNA fragments of the gene 16S. These fragments were amplified via polymerase chain reaction (PCR) using a mixture with a final volume of 15 μL, containing 5.7 μL of ddH2O, 1.5 μL of 25 mM MgCl_2_, 1.5 μL of 10 mM dNTPs (2.5mM in each dNTP), 1.5 μL of 10X amplification buffer (75 mM Tris HCl, 50 mM KCl, 20 mM (NH4) 2SO4), 1.5 μL of 2 μM solution of each of the two primers, 0.3 μL of Taq DNA polymerase 5 U / μL (Life Technologies, USA) and 1.5μL of DNA (about 30 ng / μL). The reaction conditions had a pre-heating step at 73°C for 60 s, 35 cycles of denaturation at 92°C for 10 s, primer annealing at 50°C for 35 s, and primer extension at 72°C for 90 s, followed by a final extension step of five minutes at 72°C. Prior to the sequencing reactions, PCR products were purified with Exonuclease I and Thermosensitive Alkaline Phosphatase following manufacturer recommendations (Thermo Fisher Scientific, USA) and followed ABI BigDye Terminator Cycle Sequencing Kit protocols (Life Technologies, USA), as indicated by the manufacturer. The forward primer was used in sequencing reactions, with an annealing temperature of 50°C. The resulting single-stranded products were solved in an ABI 3130xl automatic sequencer. Base calls were verified by viewing electropherograms in Geneious [32] and sequences obtained were aligned using Clustal W algorithm [33] implemented in BioEdit [34] and checked by eye.

### Candidate species delimitation

The concept of candidate species adopted in this study follows the subcategories proposed by [35]: Unconfirmed Candidate Species (UCS) corresponds to a genetically distinct lineage, for which no morphological and/or bioacoustic data are available; Deep Conspecific Lineages (DCL) represent lineages that are genetically divergent, but species cannot be differentiated by morphological and/or bioacoustic data; Confirmed Candidate Species (CCS) corresponds to a lineage that usually shows genetic divergence and can be differentiated by morphological and/or bioacoustic data, but which is not formally described as a nominal species.

#### Molecular approach

Unlike [35] and [36] that used genetic distance as molecular evidence in classifying genetic lineages, we opted for two automated barcoding algorithms as molecular evidence to delineate lineages of *Scinax*. When there was a discrepancy between the results from Automatic Barcoding Gap Discovery (ABGD) and Generalized Mixed Yule Coalescent (GMYC) for a set of lineages, we used the most conservative result, except in cases where morphological and/or bioacoustic evidence supported the less conservative approach.

The first algorithm used, ABGD [37], first estimates the genetic distance between pairs of aligned sequences from a matrix. It then statistically infers potential gaps as the minimum in the distribution of pairwise distances by dividing the sequences in a way that the distance between two sequences of different groups is greater than the distance between two sequences within a group [37]. The algorithm was performed via the web interface (http://wwwabi.snv.jussieu.fr/public/abgd/abgdweb.html) with the following priors: Kimura-two-parameters nucleotide substitution model—K2P [38], ten recursive steps, gap width of 1.0 and value of intraspecific divergence of 0.003 (3%). We opted for K2P distance because this is the most used method in barcoding analysis [39], as it effectiveness is similar to more complex models [40]. The use of a 3% divergence in the 16S rRNA in ABGD analysis has been recommended as an appropriate value for interspecific divergence when classifying lineages of Neotropical and Malagasy frogs as possible candidate species [41–42].

The magnitude of intraspecific differences can vary among lineages of anurans [41, 43]. The sole use of distance-based DNA barcoding methods can introduce errors in species delimitation [35]. To minimize these potential errors in our delimitation, we used GMYC [44] as additional algorithm based on branching patterns: branching patterns within clades reflect genetic neutral coalescing processes occurring within species [45], while branching among clusters reflect the timing of speciation events [46]. The GMYC algorithm assesses the difference in the degree of branching between these two modes of lineages evolution, through estimation of the point of greatest probability of transition between them by using likelihood-based analysis [44]. We used the single-threshold approach, which estimates a single point of transition between intra and interspecific coalescence rates [44, 47]. The GMYC was implemented in the SPLITS package (available http://r-forge.r-project.org/projects/splits) through the platform R [48]. Because the GMYC uses genealogical information rather than genetic distances, the algorithm requires an ultrametric genealogical tree as input.

We estimated an ultrametric genealogical tree using 16S rRNA sequences of 55 specimens of *Scinax* from PMIR and EBMR samples. Additionally, we selected 61 sequences available in Genbank but from species not previously assigned to any species group in the *S*. *ruber* Clade (S1 Table). As outgroup, we used seven 16S rRNA sequences from members of *Scinax rostratus* species Group, as well as *Julianus uruguayus* and *Ololygon berthae*. Ultrametric genealogical tree was estimated with BEAST software version 1.8.2 [49]. The nucleotide substitution model GTR + I + G was selected via Akaike Information Criterion [50] through jModeltest 2.1.7 [51]. The priors used for obtaining the ultrametric tree were lognormal uncorrelated relaxed clock model, substitution rate of 7.35 × 10^−3^ / site / Ma [median of ucld.mean parameter (95% HPD = 6.1–8.7 × 10^−3^)], coalescent constant size tree and random starting tree. The substitution rate used here for the 16S rRNA was obtained from [43] which inferred Bayesian genealogical dates for 216 species of Hylidae, including species of the genus *Scinax*. Three individual runs of 100 million generations each were performed in BEAST and they were sampled every 10,000 steps, totaling a posterior distribution of 10,000 trees per running. The stationarity of the posterior distributions, the effective sample size (effective sample sizes—ESS; > 200) and the convergence between runs were examined using Tracer v1.6 [52]. We combined the files containing trees after discarding the first 10% using LogCombiner v1.8.2 [49], and we built the Maximum Clade Credibility (MCC) tree using TreeAnnotator v1.8.2 [49].

In addition, we estimated interspecific pairwise distances K2P [38] between *Scinax* from PMIR and EBMR, and related species using MEGA 6.06 [53]. The distances are summarized in S2 Table.

#### Morphological approach

We used morphological data to compare our specimens with described *Scinax* species. The following diagnostic characters, all classically used in *Scinax* taxonomy, were evaluated: head shape, snout shape, skin texture, toe webbing, adult body color, and adult iris color. Additionally, ten morphometric characters were measured with digital calipers in adult specimens, according to [54]: SVL (snout-vent length), HL (head length), HW (head width), ED (horizontal eye diameter), UEW (upper eyelid width) IND (internarial distance), IOD (interorbital distance), TD (horizontal tympanum diameter), TL (tibia length) and FL (foot length). The following characters were measured according to [55]: END (eye-nostril distance), 3FD (third finger disk diameter), 4TD (fourth toe disk diameter). Length of tarsus (TAL), hand (HAL) and thigh (THL) followed [56]. Webbing formulae followed [57] as modified by [58]. Color in life was described based on field observations and color photographs of live specimens. See S3 Table for morphometric data.

#### Bioacoustic approach

Spectral and temporal parameters used in the diagnosis of *Scinax* species were obtained from 109 recordings of advertisement calls of different *Scinax* specimens taken in the study area. As the advertisement call of *Scinax* is characterized by one note we analyzed the following acoustic characteristics: note duration (s), number of pulses per note, pulse duration (s), pulse repetition rate (pulse/s), and fundamental frequency (Hz) of the note. The calls were analyzed through oscillograms and spectrograms (Blackman window, 80 Hz of frequency resolution and 1,024 data points of Discrete Fourier Transform-DFT) generated with Raven 1.5 software [59].

Bioacoustic parameters were compared with data available in the formal descriptions of the species or descriptions of advertisement calls from (or near) the type locality of each nominal species. Differences in fundamental frequency, duration of the note and pulse repetition rate are frequently used to differentiate *Scinax* species (e.g. [60–62, 10, 63]). Therefore, as opposed to [35], and following to [36], quantitative differences between calls were considered sufficient to set the CCS lineages. The advertisement call parameters from the candidate species are available in S4 Table. Advertisement call recordings were stored in the bioacoustic library of the Research Program on Biodiversity (PPBio) from Instituto Nacional de Pesquisas da Amazônia (https://ppbio.inpa.gov.br/en/home), Manaus, Brazil.

## Results

### Candidate species delimitation

**Fig 2:**
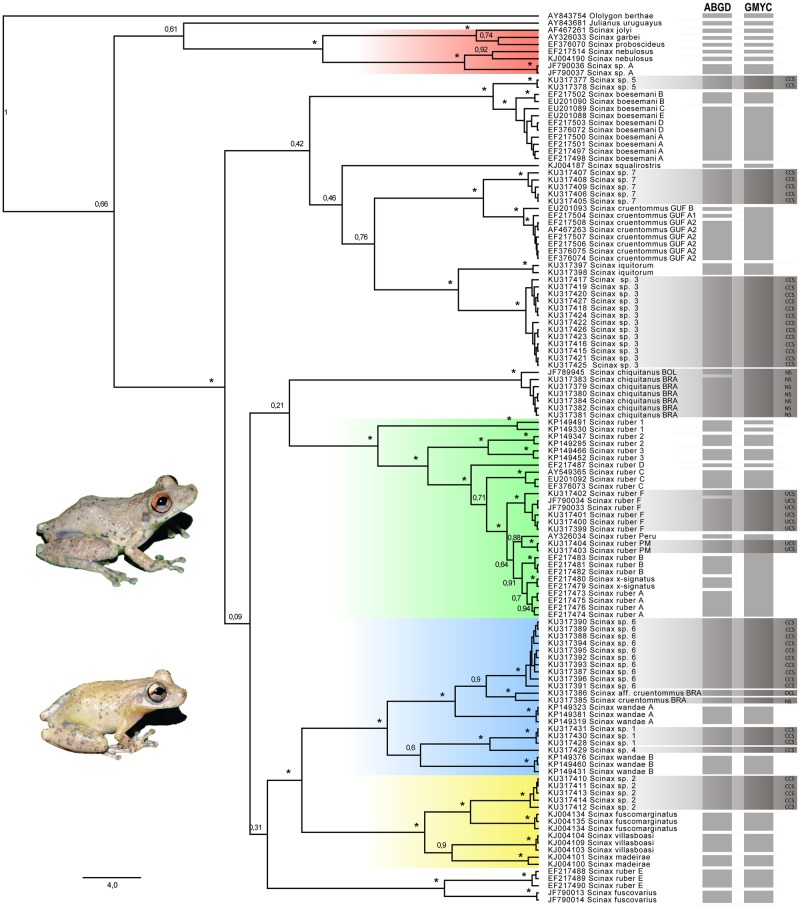
Maximum clade-credibility tree of *Scinax* from the Purus-Madeira interfluve and the east bank of the upper Madeira (Brazilian Amazonia) and correlated species. The 16S gene tree was recovered by Bayesian analyses in BEAST. Posterior probabilities are given near the nodes. Asterisks indicate PP > 0.95. Vertical gray bars indicate species delimitation with the molecular approach. Horizontal gray bars indicate species delimitation with the integrative approach. Colored areas in topology represent, from top to bottom, the *S*. *rostratus* species Group (red), *S*. *ruber* Clade (green), *S*. *wandae* Clade (blue), and *S*. *fuscomarginatus* Clade (yellow). Outgroup: members of *Scinax rostratus* species Group, plus *Julianus uruguayus* and *Ololygon berthae*. Abbreviations: ABGD, Automatic Barcoding Gap Discovery; GMYC, Generalized Mixed Yule Coalescent; CCS, Confirmed Candidate Species; UCS, Unconfirmed Candidate Species; DCL, Deep Conspecific Lineage; NS, Nominal Species.

Barcoding analysis revealed the occurrence of 12 (GMYC) and 13 (ABGD) putative units of *Scinax* in the PMIR and EBMR samples (Fig 2). GMYC and ABGD were discordant in the definition of three sets of putative units. Unlike the GMYC, the ABGD approach delineated two distinct units under the epithet *S*. *chiquitanus* (*S*. *chiquitanus* BOL and *S*. *chiquitanus* BRA). However, the GMYC-based delimitation is supported by morphology and advertisement call (see next paragraph). In addition, GMYC delimited *S*. *ruber* specimens from the central Purus-Madeira interfluve, east bank of the Madeira river and Bolivia [36] as a unique putative unity, while ABGD divided them into two distinct units. Due to lack of morphological and bioacoustic evidence, we opted for the GMYC conservative delimitation. *Scinax ruber* PM from the northern Purus-Madeira interfluve was classified as a putative unit by ABGD, differing in this from GMYC, which delimited *S*. *ruber* from Peru, *S*. *ruber* A, *S*. *ruber* B, *S*. *x-signatus* and *S*. *ruber* PM as a single putative unit. We opted for the ABGD delimitation, considering that this method reconstructed the delimitation proposed by [7], which used nuclear and mitochondrial markers. There was concordance between GMYC and ABGD for the other putative units occurring in the study area.

Morphology and/or advertisement calls were able to differentiate most of the OTUs in PMIR and EBMR. *Scinax* sp. 1 differs morphologically from *Scinax* sp. 4 (SVL, supernumerary tubercles and dorsal coloration), *Scinax* sp. 6 (SVL, relative head length, TD/ED, relative length of Toe I, shape of dentigerous processes of vomers, dorsal coloration), *S*. *wandae* A (snout shape, dorsal skin texture, dorsal coloration), *S*. *wandae* B (dorsal coloration, dorsal skin texture) and morphologically and bioacoustically from *S*. *cruentommus* (SVL, relative length of Toe I, shape of dentigerous processes of vomers, dorsal coloration; call duration, number of pulses/note, pulse repetition rate, and dominant call frequency [64–65]). *Scinax* sp. 2 can be distinguished morphologically and bioacoustically from *S*. *fuscomarginatus* (snout shape, dorsal coloration pattern of tibia; note duration and dominant call frequency; [10]) and *S*. *madeirae* (dorsal coloration pattern of tibia and infraocular; note duration and dominant call frequency); and morphologically from *S*. *villasboasi* (snout shape, relative toe length II-III, toe webbing, color pattern of dorsum and of tibia). *Scinax* sp. 3 can be distinguished from *S*. *iquitorum* by the proportion of head and foot in adult specimens, and dorsal and ventral color pattern. *Scinax* sp. 4 differs morphologically from *Scinax* sp. 6 (SVL, supernumerary tubercles, and toe webbing), *S*. *wandae* A (snout shape, dorsal skin texture, dorsal coloration), *S*. *wandae* B (dorsal coloration, dorsal skin texture) and *S*. *cruentommus* (head shape, relative length of toe III and IV, skin texture of perianal area). *Scinax* sp. 5 differs from Guiana and French Guiana specimens of *S*. *boesemani* in color pattern of dorsum and venter. *Scinax* sp. 6 differs from *S*. *wandae* A (snout shape, dorsal skin texture, dorsal coloration), *S*. *wandae B* (dorsal coloration, dorsal skin texture) and *S*. *cruentommus* (supernumerary tubercles on the finger I, outer metatarsal tubercle shape, *canthus rostralis* shape and relative length of finger discs). *Scinax* aff. *cruentommus* BRA cannot be distinguished from *S*. *cruentommus* by morphology. *Scinax ruber* F and *S*. *ruber* PM are distinguished from each other by snout shape. The morphology and advertisement call of *S*. *chiquitanus* BRA are very similar to those of *S*. *chiquitanus* BOL.

**Fig 3:**
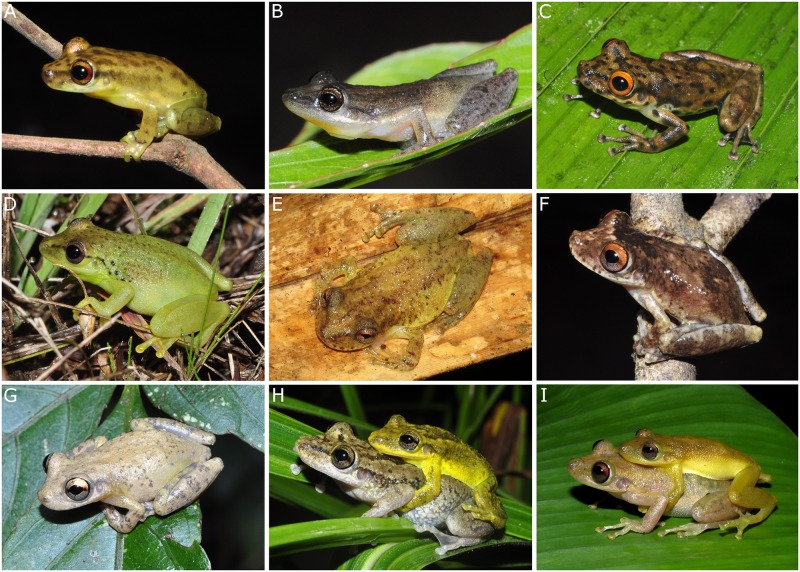
Specimens of *Scinax* from the Purus-Madeira interfluve and east bank of the upper Madeira River, Brazilian Amazonia. (A) *Scinax* sp. 1, male, SVL 20.2 mm, INPA-H 34688, from M-9. (B) *Scinax* sp. 2, male, SVL 18.1 mm, INPA-H 34667, from M-11. (C) *Scinax* sp. 3, male, SVL 31.3 mm, INPA-H 34584, from M-7. (D) *Scinax* sp. 5, male, not collected, from M-8. (E) *Scinax* sp. 6, male, SVL 25.2 mm, INPA-H 35562, from M-17. (F) *Scinax* sp. 7, male, SVL 23.9 mm, INPA-H 34623, from M-8. (G) *Scinax* aff. *cruentommus* BRA, male, SVL 25.4 mm, INPA-H 34596, from M-6. (H) *Scinax ruber* PM, couple, not collected. (I) *Scinax chiquitanus* BRA, female, SVL 33.7 mm, INPA-H 35554; male, 31.1 mm, INPA-H 35555, from M-14. Photographs by R. Fraga.

The integration of molecular, morphological and bioacoustic evidence allowed the delineation of more species of *Scinax* than there are available epithets from the PMIR and EBMR. Our integrative analysis revealed seven CCS lineages (*Scinax* sp. 1–7), two UCS (*Scinax ruber* F and *S*. *ruber* PM), one DCL (*Scinax* aff. *cruentommus* BRA) and only two nominal species (*S*. *chiquitanus* BRA and *S*. *cruentommus* BRA) (Fig 3). *Scinax ruber* PM (UCS), *S*. aff. *cruentommus* BRA (DCL) and the seven CCS lineages delimited in this study were unknown until the current study. *Scinax chiquitanus* and *S*. *ruber* F are recorded for the first time in Brazil. If CCS and UCS are considered as lineages that represent undescribed species, 82% of the *Scinax* species richness in the PMIR and EBMR is not described, an increase of 450% in the currently-known regional species richness. More broadly, taking into account the lineages that are not associated with any species group occurring in the Brazilian Amazonia, 30% of the overall *Scinax* species richness revealed in this study is not formally described, representing an increase of 43% in the number of *Scinax* species currently-known.

Our 16S gene tree strongly supports three major clades where most of the OTUs from PMIR and EBMR were included. The *Scinax wandae* Clade comprises *S*. *cruentommus* BRA, *S*. aff. *cruentommus* BRA, *S*. *wandae* A, *S*. *wandae* B, *Scinax* sp. 1, *Scinax* sp. 4 and *Scinax* sp. 6. Pairwise genetic distances within the *S*. *wandae* Clade range from 2 to 14%, with the shortest distance recorded between *S*. aff. *cruentommus* BRA and. *S*. *cruentommus* BRA (2%). The *Scinax fuscomarginatus* Clade contains *S*. *fuscomarginatus*, *S*. *madeirae*, *S*. *villasboasi* and *Scinax* sp. 2. The pairwise genetic distances between *Scinax* sp. 2 and other members of this clade range from 8 to 12%. The *Scinax wandae* Clade and *S*. *fuscomarginatus* Clade are reciprocally monophyletic. *Scinax ruber* F and *S*. *ruber* PM were included within the highly supported clade containing *S*. *ruber* A–D, *S*. *ruber* Peru, and *S*. *x-signatus*. In turn, this clade is the sister of *S*. *ruber* 2 + *S*. *ruber* 3 and forms a major clade with *S*. *ruber* 1, the latter in basal position. Pairwise genetic distances among *S*. *ruber* F and *S*. *ruber* PM from other *S*. *ruber* OTUs (except *S*. *ruber* E) range from 2 to 11%. *Scinax ruber* E is placed as the sister of *S*. *fuscovarius* and so questions the monophyly of the currently recognized *S*. *ruber* OTUs. The distances between *S*. *ruber* E and other *S*. *ruber* OTUs range from 18 to 29%.

## Discussion

### Barcoding methods

We used the algorithms ABGD and GMYC to define *Scinax* candidate species in this study. Despite differences, in most cases, there was a convergence of both algorithms in delimitating *Scinax* candidate species from the study area. For the data set involving all putative units in the MCC tree, and using the candidate species proposed by [7, 66] as a reference (delimitated by using more than one molecular marker), ABGD showed greater accuracy in defining the candidate species when compared to GMYC, as this letter method tended to group two or more candidate species.

The incongruity between the results from ABGD and GMYC obtained in this study may be explained by the quality characteristics of the dataset quality. Although GMYC is a robust algorithm such conditions as the presence of gaps, large number of singletons and low species richness [67], the algorithm is sensitive to small genetic differences between species [68], which have been found between most of the species in the *S*. *ruber* Clade [7] [this study]. In turn, the main factor influencing the ABGD delimitation is the value of interspecific genetic divergence defined *a priori* [37], which requires existing knowledge of the target group. Despite the differences in the GMYC and ABGD delimitations, results highlight the importance of the use of both these barcoding algorithms as molecular evidence in integrative analyses. The use of just one algorithm in our data set would have given less accurate lineage delimitation.

### Species richness and conservation

We used for the first time integrated molecular, morphological and bioacoustic data to evaluate species richness of *Scinax* treefrogs at a regional scale in Amazonia. Like other studies that have used an integrative approach to address frog richness in this biome (*e*.*g*. [2, 5, 36, 69]), our results show that the knowledge of the species diversity of Amazonian frogs is highly inadequate. The underestimation of Amazonian species of *Scinax* (30%) is similar to the percentage of unnamed amphibian species proposed by [70] for the whole Neotropics (39%). However, at a regional scale (PMIR and EBMR), the proportion of unidentified *Scinax* species is more than twice as high (82%) as the estimates for the Neotropics. The increase in the known *Scinax* species richness from the PMIR and EBMR (450%) is the highest increase in Amazonian frog diversity ever reported, being greater than estimates proposed in studies involving the genera *Engystomops* (150–250% [5]), *Hypsiboas* (200–350% [5]), *Osteocephalus* (37.5%–300% [3, 69]), and *Pristimantis* (200% [71]). Despite the cryptic diversity [7, 21] and the complex taxonomic history of several *Scinax* species, the high proportion of undescribed species found in the region of PMIR and EBMR is mainly due to (1) integration of different lines of evidence in the species identification process, and (2) the fact that the region represents one of the most poorly-studied areas in Brazilian Amazonia [72]. Our results suggest that further integrative studies of anurans from poorly investigated parts of the biome may significantly contribute to an improved knowledge of the real anuran diversity of the region and improve our understanding of the overall biodiversity of the Neotropics.

Unfortunately, most of the study area is threatened by infrastructure development associated with human settlements. The PMIR is intersected by the BR-319 federal highway. Although it was partially abandoned a few years after its construction in 1973 [22], the presence of the highway facilitated the process of deforestation in the region, coming especially from the state of Rondônia [22]. Currently, the BR-319 is being repaired and resurfaced, and modeling studies have predicted the resulting deforestation of up to 5.4 million hectares by 2050, which represents approximately one-third of the total area of the interfluve [23–24]. In addition to the imminent threat of reconstruction of the BR-319, the construction of two large hydroelectric projects on the southern PMIR and EBMR (Porto Velho, Rondônia) also threatens the high biodiversity of the area [22]. This is an alarming scenario for the conservation of local *Scinax* populations, considering that only two CCS lineages (*Scinax* sp. 2 and *S*. sp. 5) and two UCS lineages (*Scinax ruber* F and *S*. *ruber* PM) are known to be tolerant of habitat disturbance. Species with small geographic ranges are usually more susceptible to anthropogenic threats than widely distributed species [73–74]. Despite the high sampling effort, two CCS lineages (*Scinax* sp. 1, *S*. sp. 4) that inhabit the forested habitats were found in only one locality in the PMIR. The only sampling module in which *Scinax* sp. 4 was registered is currently under heavy pressure from illegal logging (M. Ferrão, personal communication). In addition, a rapid increase of deforestation of the northern region of the BR-319 is predicted by [24]. Considering a combination of current anthropic disturbance and limited range, *Scinax* sp. 4 may be classified as an endangered species.

Description of new species may contribute to identification of priority areas for biodiversity conservation [75], which potentially results in protection and management of natural resources [76]. Contrarily, lack of formal description of new species has led to neglect hundreds of species when mapping priority areas for conservation (e.g. [77]). Since formally named species are important for habitat and biodiversity assessment, especially in such threatened landscapes as the study area, descriptions of the seven CCS disclosed here will be the subject of our future papers.

### Systematic remarks

Contrary to [17], this study did not place *Julianus* Duellman, Marion and Hedges, 2016 in a sister position to the genus *Scinax*. In our 16S gene tree, *Julianus uruguayus* (Schmidt, 1944) represents a sister lineage to the *Scinax rostratus* species Group (with low support), and together with it forms a sister clade to all remaining species of *Scinax* (with low support). This arrangement may be an artifact of our use of only one locus in our analyses. On the other hand, our 16S gene tree strongly supports the *S*. *rostratus* species Group as a monophyletic unit, as previously recognized by [18, 78–79]. Additionally, most of our OTUs were placed in three major clades (*S*. *fuscomarginatus*, *S*. *ruber*, *S*. *wandae*) by our phylogenetic analyses. Strong Bayesian support for both clades may be interpreted as evidence of species grouping. Nevertheless, a more extensive sampling and an appropriate morphological and phylogenetic approach will be required to better delimitate individual species groups.

*Scinax cruentommus* has been widely reported from Peru, Brazil, Ecuador and Colombia [65], and from French Guiana [7, 42, 80–82]. In our barcoding analyses, a *Scinax* specimen from PMIR identified as *S*. *cruentommus* (*S*. *cruentommus* BRA) differs from *S*. *cruentommus* GUF (*sensu* [7, 42, 80–81]) with genetic distances of 31 to 32%. Unlike *S*. *cruentommus* BRA, specimens of *S*. *cruentommus* GUF do not possess a horizontal red bar in the iris (see Figure 5 in [81]), which [64] proposed as an important diagnostic character of *S*. *cruentommus*. Moreover, notable differences between advertisement call of *S*. *cruentommus* from the upper Negro river (Amazonas, Brazil) and *S*. *cruentommus* from French Guiana (*sensu* [82]) were reported by [65]. Based on this evidence, and in the proposition that widely-distributed small-sized frogs may potentially represent species complexes [2, 5, 7, 21, 43, 71, 83], we argue that there is likely to be more than one species associated with the name *S*. *cruentommus*, and that specimens from French Guiana called *S*. *cruentommus* represent a different, and undescribed, taxon.

*Scinax ruber* F and *S*. *ruber* PM from PMIR and EBMR placed in the *Scinax ruber* Clade, as did *S*. *ruber* 1–3 from Colombia (in basal position). Although there is no doubt about the validity of the name *S*. *ruber* [19], the strong variance in pairwise genetic distance between all OTUs in this clade plus *S*. *ruber* E (1% to 29%) suggests cryptic diversity [7, 66] [this study], as well as misidentification (perhaps for *S*. *ruber* E and *S*. *x-signatus* from French Guiana). The different *Scinax* forms which this name has been applied are distributed over a wide geographic area (Brazil, French Guiana, Surinam, Colombia, Ecuador, Peru, Bolivia) indicating the need for a thorough revision. Investigation of morphological, bioacoustic and genetic characteristics of *S*. *ruber* from its neotype locality (Paramaribo, Surinam) might be the first step in resolving this taxonomic problem. This should then be followed by a collaborative international effort to clarify the taxonomic status and the evolutionary relationships of different OTUs related to *S*. *ruber*.

## Supporting Information

S1 Appendix Formulae for cryptic diversity estimates. (PDF)

S2 Appendix List of specimens examined for the morphological comparisons. (PDF)

S1 Table Specimens examined, voucher numbers, localities, and GenBank accession numbers. (PDF)

S2 Table Genetic divergence between *Scinax* species from the Purus-Madeira interfluve and east bank of the Upper Madeira river and related species. (PDF)

S3 Table Morphometric data of *Scinax* species from the Purus-Madeira interfluve and east bank of the upper Madeira river, Brazilian Amazonia. (PDF)

S4 Table Temporal and spectral parameters of the advertisement call of *Scinax* species from the Purus-Madeira interfluve and east bank of the upper Madeira river, Brazilian Amazonia. (PDF)

## References

[1] Stuart SN, Chanson JS, Cox NA, Young BE, Rodrigues ASL, Fischman DL, et al Status and trends of amphibian declines and extinctions worldwide. Science. 2004; 306: 1783–1786. 10.1126/science.1103538 15486254

[2] Fouquet A, Cassini CS, Haddad CFB, Pech N, Rodrigues MT. Species delimitation, patterns of diversification and historical biogeography of theNeotropical frog genus *Adenomera* (Anura, Leptodactylidae). J Biogeogr. 2013; 41(5): 855–870. 10.1111/jbi.12250

[3] Jungfer KH, Faivovich J, Padial JM, Castroviejo-Fisher S, Lyra MM, Berneck B, et al Systematics of spiny-backed treefrogs (Hylidae: *Osteocephalus*): An Amazonian puzzle. Zool Scr. 2013; 42: 351–380. 10.1111/zsc.12015

[4] Fouquet A, Loebmann D, Castroviejo-Fisher S, Padial JM, Orrico VGD, Lyra ML, et al From Amazonia to the Atlantic forest: Molecular phylogeny of Phyzelaphryninae frogs reveals unexpected diversity and a striking biogeographic pattern emphasizing conservation challenges. Mol Phylogenet Evol. 2012; 65(2): 547–561. 10.1016/j.ympev.2012.07.012 22842094

[5] Funk WC, Caminer M, Ron SR. High levels of cryptic species diversity uncovered in Amazonian frogs. Proc R Soc Lond B Biol Sci. 2012; 279: 1806–1814. 10.1098/rspb.2011.1653 22130600 PMC3297442

[6] Caminer MA, Ron SR. Systematics of treefrogs of the *Hypsiboas calcaratus* and *Hypsiboas fasciatus* species complex (Anura, Hylidae) with the description of four new species. Zookeys. 2014; 370: 1–68. 10.3897/zookeys.370.6291 24478591 PMC3904076

[7] Fouquet A, Vences M, Salducci MD, Meyer A, Marty C, Gilles A. Revealing cryptic diversity using molecular phylogenetics and phylogeography in frogs of the *Scinax ruber* and *Rhinella margaritifera* species groups. Mol Phylogenet Evol. 2007a; 43: 567–582. 10.1016/j.ympev.2006.12.006 17303441

[8] Pombal JPJ, Bastos RP, Haddad CFB. Vocalizações de algumas espécies do gênero *Scinax* (Anura, Hylidae) do Sudeste do Brasil e comentários taxonômicos. Naturalia. 1995; 20: 213–225.

[9] Nunes I, Kwet A, Pombal JPJ. Taxonomic revision of the *Scinax alter* species complex (Anura: Hylidae). Copeia. 2012; 2012(3): 554–569. 10.1643/CH-11-088

[10] Brusquetti F, Jansen M, Barrio-Amarós C, Segalla M, Haddad CFB. Taxonomic review of *Scinax fuscomarginatus* (Lutz, 1925) and related species (Anura; Hylidae). Zool J Linn Soc. 2014; 171: 783–821. 10.1111/zoj.12148

[11] Padial JM, De la Riva I. Integrative taxonomy reveals cryptic Amazonian species of *Pristimantis* (Anura). Zool J Linn Soc. 2009; 155: 97–122. 10.1111/j.1096-3642.2008.00424.x

[12] Simões PI, Lima AP, Farias I. The description of a cryptic species related to the pan-Amazonian frog *Allobates femoralis*. Zootaxa. 2010; 2406: 1–28.

[13] Simões PI, Kaefer IL, Farias IP, Lima AP. An integrative appraisal of the diagnosis and distribution of *Allobates sumtuosus* (Morales, 2002) (Anura, Aromobatidae). Zootaxa. 2013; 3746: 401–421. 10.11646/zootaxa.3746.3.1 25113485

[14] Lima AP, Simões PI, Kaefer IL. A new species of *Allobates* (Anura: Aromobatidae) from the Tapajós River basin, Pará State, Brazil. Zootaxa. 2014; 3889(3): 355–387. 10.11646/zootaxa.3980.4.3 25544274

[15] Lima AP, Simões PI, Kaefer IL. A new species of *Allobates* (Anura: Aromobatidae) from Parque Nacional da Amazônia, Pará State, Brazil. Zootaxa. 2015; 3980(4): 501–525. 10.11646/zootaxa.3980.4.3 26249969

[16] Kaefer IL, Tsuji-Nishikido BM, Mota EP, Farias IP, Lima AP. The early stages of speciation in Amazonian forest frogs: phenotypic conservatism despite strong genetic structure. Evol Biol. 2013; 40: 228–245. 10.1007/s11692-012-9205-4

[17] Duellman WE, Marion AB, Hedges SB. Phylogenetics, Classification, and Biogeography of the Treefrogs (Amphibia: Anura: Arboranae). Zootaxa. 2016; 4104(1): 1–109. 10.11646/zootaxa.4104.1.1 27394762

[18] Faivovich J, Haddad CFB, Garcia PCA, Frost DR, Campbell JA, Weeler WC. Systematic review of the frog family Hylidae, with special reference to Hylinae: phylogenetic analysis and taxonomic revision. Bulletin of the American Museum of Natural History. 2005; 294; 1–240. 10.1206/0003-0090(2005)294[0001:SROTFF]2.0.CO;2

[19] Sturaro MJ, Peloso PLV. A new species of *Scinax* Wagler, 1830 (Anura: Hylidae) from the middle Amazon River Basin, Brazil. Pap Avulsos Zool. 2014; 54(2): 9–23. 10.1590/0031-1049.2014.54.02

[20] Nogueira L, Solé M, Siqueira S, Affonso PRAM, Strüssmann C, Sampaio I. Genetic analysis reveals candidate species in the *Scinax catharinae* Clade (Amphibia: Anura) from Central Brazil. Genet Mol Biol. 2016; 39(1): 49–53. 10.1590/1678-4685-GMB-2015-0037 27007898 PMC4807394

[21] Menezes L, Canedo C, Batalha-Filho H, Garda AA, Gehara M, et al Multilocus Phylogeography of the Treefrog *Scinax eurydice* (Anura, Hylidae) Reveals a Plio-Pleistocene Diversification in the Atlantic Forest. PLoS One. 2016; 11(6): e0154626 10.1371/journal.pone.0154626 27248688 PMC4889069

[22] Fearnside PM, Graça PMLA. BR-319: Brazil’s Manaus-Porto Velho Highway and the potential impact of linking the arc of deforestation to central Amazonia. Environ Manage. 2006; 38(5): 705–716. 10.1007/s00267-005-0295-y 16990982

[23] Soares-Filho BS, Nepstad DC, Curran LM, Cerqueira GC, Garcia RA, Azevedo-Ramos C, et al Modelling conservation in the Amazon basin. Nature. 2006; 440:520–523. 10.1038/nature04389 16554817

[24] Maldonado FD, Keizer EWH, Graça PMLA, Fearnside PM, Vitel CS. (2012). Previsão temporal da distribuição espacial do desmatamento no interflúvio Purus-Madeira até o ano 2050 In Sousa-Junior WC, Waichman AV, Sinisgalli PAA, Angelis CF, Romeiro AR, editors. Rio Purus: Água, Território e Sociedade na Amazônia Sul-Ocidental. Ecuador: LibriMundi Press; 2012 pp. 183–196.

[25] Sombroek W. Amazon landforms and soils relation to biological diversity. Acta Amazon. 2000; 30: 81–100.

[26] Cintra BBL, Schietti J, Emilio T, Martins D, Moulatlet G, Souza P, et al Soil physical restrictions and hydrology regulate stand age and wood biomass turnover rates of Purus—Madeira interfluvial wetlands in Amazonia. Biogeosciences. 2013; 10: 7759–7774. 10.5194/bg-10-7759-2013

[27] Rosetti DF, Toledo PM, Góes AM. New geological framework for Western Amazonia (Brazil) and implications for biogeography and evolution. Quat Res. 2015; 63: 78–89. 10.1016/j.yqres.2004.10.001

[28] IBGE Instituto Brasileiro de Geografia e Estatística. Recursos naturais e meio ambiente: uma visão do Brasil. Rio de Janeiro: IBGE; 1997.

[29] Magnusson WE, Braga-Neto R, Pezzini F, Baccaro F, Bergallo H, Penha J, et al Biodiversidade e monitoramento ambiental integrado: o sistema rapeld na Amazônia. Santo André: Publisher Attema; 2013.

[30] Campbell HW, Christman SP. Field techniques for herpetofaunal community analysis In Scott NJ Jr, editor. Herpetological Communities: a Symposium of the Society for the Study of Amphibians and Reptiles and the Herpetologists`League. Washington: U.S. Fish Wild. Serv; 1982 pp. 193–200.

[31] Palumbi SR, Martin AP, Romano SL, McMillan WO, Stice L, Grabowski G. The sample tool’s guide to PCR. Honolulu: University of Hawaii; 1991.

[32] Kearse M, Moir R, Wilson A, Stones-Havas S, Cheung M, Sturrock S, et al Geneious Basic: An integrated and extendable desktop software platform for the organization and analysis of sequence data. Bioinformatics. 2012; 28(12): 1647–1649. 10.1093/bioinformatics/bts199 22543367 PMC3371832

[33] Thompson JD, Higgins DG, Gibson TJ. Clustal W: improving the sensitivity of progressive multiple sequence alignment through sequence weighting, position-specific gap penalties and weight matrix choice. Nucleic Acids Res. 1994; 22(22): 4673–4680. 10.1093/nar/22.22.4673 7984417 PMC308517

[34] Hall TA. BioEdit: a user-friendly biological sequence alignment editor and analysis program for Windows 95/98/NT. Nucleic Acids Symp Ser. 1999; 41: 95–98.

[35] Vieites DR, Wollenberg KC, Andreone F, Köhler J, Glaw F, Vences M. Vast underestimation of Madagascar’s biodiversity evidenced by an integrative amphibian inventory. Proc Natl Acad Sci U S A. 2009; 106(20): 8267–72. 10.1073/pnas.0810821106 19416818 PMC2688882

[36] Jansen M, Bloch R, Schulze A, Pfenninger M. Integrative inventory of Bolivia’s lowland anurans reveals hidden diversity. Zool Scr. 2011; 40: 567–583. 10.1111/j.1463-6409.2011.00498.x

[37] Puillandre N, Lambert A, Brouillet S, Achaz G. ABGD, Automatic Barcode Gap Discovery for primary species delimitation. Mol Ecol. 2012; 21: 1864–1877. 10.1111/j.1365-294X.2011.05239.x 21883587

[38] Kimura M. A simple method for estimating evolutionary rate of base substitutions through comparative studies of nucleotide sequences. J Mol Evol. 1980; 16: 111–120. 7463489 10.1007/BF01731581

[39] Ward RD. DNA barcode divergence among species and genera of birds and fishes. Mol Ecol Resour. 2009; 9: 1077–1085. 10.1111/j.1755-0998.2009.02541.x 21564845

[40] Collins RA, Boykin LM, Cruickshank RH, Armstrong KF. Barcoding's next top model: an evaluation of nucleotide substitution models for specimen identification. Methods Ecol Evol. 2012; 3: 457–465. 10.1111/j.2041-210X.2011.00176.x

[41] Vences M, Thomas M, van der Meijden A, Chiari Y, Vieites D. Comparative performance of the 16S rRNA gene in DNA barcoding of amphibians. Front Zool. 2005; 2: 5 10.1186/1742-9994-2-5 15771783 PMC555853

[42] Fouquet A, Gilles A, Vences M, Marty C, Blanc M, Gemmell NJ. Underestimation of species richness in neotropical frogs revealed by mtDNA analyses. PloS One. 2007b; 10: e1109 10.1371/journal.pone.0001109 17971872 PMC2040503

[43] Gehara M, Crawford AJ, Orrico VGD, Rodríguez A, Lötters S, Fouquet A, et al High levels of diversity uncovered in a widespread nominal taxon: continental phylogeography of the Neotropical treefrog *Dendropsophus minutus*. PloS One. 2014; 9(9): e103958 10.1371/journal.pone.0103958 25208078 PMC4160190

[44] Pons J, Barraclough TG, Gomez—Zurita J. Sequence based species delimitation for the DNA taxonomy of undescribed insects. Syst Biol. 2006; 55: 595–609. 10.1080/10635150600852011 16967577

[45] Kingman JFC. The coalescent. Stoch Process Their Appl. 1982; 13: 235–248. 10.1016/0304-4149(82)90011-4

[46] Yule GU. A mathematical theory of evolution based on the conclusions of Dr. J. C. Willis, F.R.S. Philos Trans R Soc Lond B Biol Sci. 1924; 213: 21–87. 10.1098/rstb.1925.0002

[47] Fujisawa T, Barraclough TG. Delimiting species using single-locus data and the Generalized Mixed Yule Coalescent approach: a revised method and evaluation on simulated data sets. Syst Biol. 2013; 65: 707–724. 10.1093/sysbio/syt033 23681854 PMC3739884

[48] R Core Team. R: A language and environment for statistical computing. Vienna, Austria: R Foundation for Statistical Computing 2016 Available: https://cran.r-project.org/doc/manuals/r-release/fullrefman.pdf

[49] Drummond AJ, Suchard MA, Xie D, Rambaut A. Bayesian phylogenetics with BEAUti and the BEAST 1.7. Mol Biol Evol. 2012; 29: 1969–1973. 10.1093/molbev/mss075 22367748 PMC3408070

[50] Akaike H. A new look at the statistical model identification. IEEE Trans Automat Contr. 1974; 19(6): 716–723.

[51] Darriba D, Taboada GL, Doallo R, Posada D. jModelTest 2: more models, new heuristics and parallel computing. Nat Methods. 2012; 9(8): 772 10.1038/nmeth.2109 22847109 PMC4594756

[52] Rambaut A, Suchard MA, Drummond AJ. Tracer v1.6. 2014. Available: http://beast.bio.ed.ac.uk/Tracer.

[53] Tamura K, Stecher G, Peterson D, Filipski A, Kumar S. MEGA6: Molecular Evolutionary Genetics Analysis version 6.0. Mol Biol Evol. 2013; 30: 2725–2729. 10.1093/molbev/mst197 24132122 PMC3840312

[54] Duellman WE. The hylid frogs of Middle America 1. 1st ed Lawrence: University of Kansas 1970

[55] Napoli MF. A new species of allied to *Hyla circumdata* (Anura: Hylidae) from Serra da Mantiqueira, southeastern Brazil. Herpetologica. 2005; 61: 63–69. 10.1655/03-41

[56] Heyer WR, Rand AS, Cruz CAG, Peixoto OL, Nelson CE. Frogs of Boracéia. Arquivos de Zoologia. 1990; 31: 231–410.

[57] Savage JM, Heyer WR. Variation and distribution in the tree-frog genus *Phyllomedusa* in Costa Rica, Central America. Stud Neotrop Fauna Environ. 1967; 5: 111–131.

[58] Myers CW, Duellman WE. A new species of *Hyla* from Cerro Colorado, and other tree frog records and geographical notes from Western Panama. Am Mus Novit. 1982; 2752: 1–32.

[59] Bioacoustics Research Program. Raven Pro: Interactive Sound Analysis Software. Ver. 1.5. Ithaca: The Cornell Lab of Ornithology: 2015.

[60] Duellman WE, Wiens JJ. Hylid frogs of the genus *Scinax* Wagler, 1830, in Amazonian Ecuador and Peru. Occas. pap. Mus. Nat. Hist. (Lawrence). 1993; 153: 1–57.

[61] Nunes I, Carvalho RRJ, Pereira EG. A new species of *Scinax* Wagler (Anura: Hylidae) from Cerrado of Brazil. Zootaxa. 2010; 2514: 24–34.

[62] Nunes I, Pombal JPJ. A new snouted treefrog of the speciose genus *Scinax* Wagler (Anura, Hylidae) from northeastern Brazil. Herpetologica. 2011; 67(1): 80–88. 10.1655/HERPETOLOGICA-D-10-00026.1

[63] Juncá FA, Napoli MF, Nunes I, Mercês EA, Abreu RO. A New Species of the *Scinax ruber* Clade (Anura, Hylidae) from the Espinhaço Range, Northeastern Brazil. Herpetologica. 2015; 71(4): 299–309. 10.1655/HERPETOLOGICA-D-14-00032

[64] Duellman WE. A new species of *Hyla* from Amazonian Ecuador. Copeia. 1972; 1972: 265–271.

[65] Carvalho TR, Teixeira BFV, Duellman WE, Giaretta A. *Scinax cruentommus* (Anura: Hylidae) in the upper Rio Negro drainage, Amazonas state, Brazil, with the redescription of its advertisement call. Phyllomedusa. 2015; 14(2): 139–146. 10.11606/issn.2316-9079.v14i2p139-146

[66] Guarnizo CE, Paz A, Muñoz-Ortiz A, Flechas SV, Méndez-Narvaéz J, Crawford AJ. DNA barcoding survey of anurans across the Eastern Cordillera of Colombia and the impact of the Andes on cryptic diversity. PloS One. 2015; 10(5): e0127312 10.1371/journal.pone.0127312 26000447 PMC4441516

[67] Talavera G, Dinca V, Vila R. Factors affecting species delimitations with the GMYC model: insights from a butterfly survey. Methods Ecol Evol. 2013; 4: 1101–1110. 10.1111/2041-210X.12107

[68] Zhang J, Kapli P, Pavlidis P, Stamatakis A. A general species delimitation method with applications to phylogenetic placements. Bioinformatics. 2013; 29: 2869–2876. 10.1093/bioinformatics/btt499 23990417 PMC3810850

[69] Ron SR, Venegas PJ, Toral E, Read M, Ortiz DA, Manzano AL. Systematics of the *Osteocephalus buckleyi* species complex (Anura: Hylidae) from Ecuador and Peru. ZooKeys. 2012; 229: 1–52. 10.3897/zookeys.229.3580 23166473 PMC3494004

[70] Giam X, Scheffers BR, Sodhi NS, Wilcove DS, Ceballos G, Ehrlich PR. Reservoirs of richness: least disturbed tropical forest are centers of undescribed species diversity. Proc R Soc Lond B Biol Sci. 2011; 279: 67–76. 10.1098/rspb.2011.0433 21593037 PMC3223638

[71] Elmer KR, Dávila JA, Lougheed SC. Cryptic diversity and deep divergence in an upper Amazonian leaflitter frog, *Eleutherodactylus ockendeni*. BMC Evol Biol. 2007; 7: 247 10.1186/1471-2148-7-247 18154647 PMC2254618

[72] Peloso P.L.V. A safe place for amphibians? A cautionary tale on the taxonomy and conservation of frogs, caecilians, and salamanders in the Brazilian Amazonia. Zoologia (Curitiba). 2010; 27(5); 667–673. 10.1590/S1984-46702010000500001

[73] Stuart BL, Inger RF, Voris HK. High level of cryptic species diversity revealed by sympatric lineages of Southeast Asian forest frogs. Biol Lett. 2006; 2: 470–475. 10.1098/rsbl.2006.0505 17148433 PMC1686201

[74] Toledo LF, Batista RF. Integrative Study of Brazilian Anurans: geographic Distribution, Size, Environment, Taxonomy, and Conservation. Biotropica. 2012; 44(6): 785–792. 10.1111/j.1744-7429.2012.00866.x

[75] Köhler J, Vieites DR, Bonett RM, Garcia FH, Glaw F, Steinke D, et al New amphibians and global conservation: A boost in species discoveries in a highly endangered vertebrate group. Bioscience. 2005; 55: 693–696.

[76] Bickford D, Lohman DJ, Sodhi NS, Ng PKL, Meier R, Winker K, et al Cryptic species as a window on diversity and conservation. Trends Ecol Evol. 2007; 22(3): 148–155. 10.1016/j.tree.2006.11.004 17129636

[77] Jenkins CN, Pimm SL, Joppa LN. Global patterns of terrestrial vertebrate diversity and conservation. Proc Natl Acad Sci U S A. 2013; 110(28): E2602–10. 10.1073/pnas.1302251110 23803854 PMC3710798

[78] Faivovich J. A cladistic analysis of *Scinax* (Anura: Hylidae). Cladistics. 2002; 18(4): 367–393. 10.1111/j.1096-0031.2002.tb00157.x 34911217

[79] Wiens JJ, Kuczynski CA, Hua X, Moen DS. An expanded phylogeny of treefrogs (Hylidae) based on nuclear and mitochondrial sequence data. Mol Phylogenet Evol. 2010; 55: 871–882. 10.1016/j.ympev.2010.03.013 20304077

[80] Salducci MD, Marty C, Chappaz R, Gilles A. Molecular phylogeny of French Guiana Hylinae: implications for the systematic and biodiversity of the neotropical frogs. C R Biol. 2002; 325: 141–153. 10.1016/S1631-0691(02)01423-3 11980175

[81] Salducci MD, Marty C, Fouquet A, Gilles A. Phylogenetic relationships and biodiversity in Hylids (Anura: Hylidae) from French Guiana. C R Biol. 2005; 328: 1009–1024. 10.1016/j.crvi.2005.07.005 16286090

[82] Lescure J, Marty C. Atlas des amphibiens de Guyane. Patrimoines Naturales. 2000; 45: 1–388.

[83] Ortega-Andrade HM, Rojas-Soto OR, Valencia JH, Espinosa de los Monteros A, Morrone JJ, Ron SR, et al Insights from integrative systematics reveal cryptic diversity in *Pristimantis* frogs (Anura: Craugastoridae) from the Upper Amazon Basin. Plos One. 2015; 10(11): e0143392 10.1371/journal.pone.0143392 26600198 PMC4658055

