# Supplementary material for: High Species Richness of Scinax Treefrogs (Hylidae) in a Threatened Amazonian Landscape Revealed by an Integrative Approach
Source: PLoS One. 2016 Nov 2;11(11):e0165679. doi: 10.1371/journal.pone.0165679 (PMC5091857; doi:10.1371/journal.pone.0165679)
Supplement: S1 Appendix — (PDF) [file pone.0165679.s001.pdf]

Formulae for cryptic diversity estimates

The calculation of cryptic diversity estimates (underestimation and/or increasement of species) is not standardized among studies focused on Neotropical diversity of frogs. Therefore, comparisons between diversity estimates may be difficult among different studies. Here, we suggest a standardization in the use of the terms underestimation and increasement in biodiversity studies. Furthermore, we encourage researchers to use these formulas in future comparisons.

We use data from *Engystomops* presented by [1] to show how the formulas work. After integrative analyses, two currently recognized species of *Engystomops* represent actually five to seven species. We use the conservative result (two nominal species + three candidate species) in our examples.

Increase formula

UND = (*Ce* * *100*)/*De*

Where *Ce* is the candidate species number in a sampled area, *De* is the number of nominal species in the same area, and the number *100* is a fixed percentage.

Example:

UND = ((3 * 100)/2) = **150**% of increase.

Underestimation formula

INC = (*Ce* * *100*)/(*De* + *Ce*)

Where *Ce* is the candidate species number in a sampled area, *De* is the number of nominal species in the same area, and the number *100* is a fixed percentage.

Example:

INC = (3 * 100)/(2+3) = **60**% of underestimation.

References

1. Funk WC, Caminer M, Ron SR. High levels of cryptic species diversity uncovered in Amazonian frogs. Proc R Soc Lond B Biol Sci. 2012; 279: 1806-1814. doi: 10.1098/rspb.2011.1653
