# Supplementary material for: High Species Richness of Scinax Treefrogs (Hylidae) in a Threatened Amazonian Landscape Revealed by an Integrative Approach
Source: PLoS One. 2016 Nov 2;11(11):e0165679. doi: 10.1371/journal.pone.0165679 (PMC5091857; doi:10.1371/journal.pone.0165679)
Supplement: S2 Appendix — (PDF) [file pone.0165679.s002.pdf]

List of specimens examined for morphological comparisons. Abbreviations. - AM, state highway in Amazonas, Brazil. - BR-319, federal highway, Brazil. - PDBFF, Projeto Dinâmica Biológica de Fragmentos Florestais (a project in Brazil focused on dynamics of forest fragments.). - km, kilometer. - UHE, hydroelectric plant (usina hidrelétrica in Portuguese). - INPA-H, herpetological section of the zoological collections of the Instituto Nacional de Pesquisas da Amazônia, Manaus, Brazil. - APL, Laboratory of Population Ecology of Instituto Nacional de Pesquisas da Amazônia, Manaus, Brazil. - RMNH, Nationaal Natuurhistorisch Museum, Leiden, The Netherlands. - QCAZ, Museo de Zoología, Pontifica Universidad Católica del Ecuador, Quito, Ecuador. - KU, University of Kansas, Museum of Natural History, Division of Herpetology, Lawrence, Kansas, USA. – ANDES-A, Museo de Historia Natural ANDES, Universidad de los Andes, Bogotá, Colombia.

***Scinax* sp. 1:** BRAZIL: *AMAZONAS*: Tapauá, BR-319, km 450 (INPA-H 34688, INPA-H 34691, INPA-H 34689, INPA-H 34692, INPA-H 34690, INPA-H 34700).

***Scinax* sp. 2**: BRAZIL: *AMAZONAS*: Humaitá, BR-319, km 620 (INPA-H 34651, INPA-H 34657, INPA-H 34664, INPA-H 34666, INPA-H 34667, INPA-H 34668, INPA-H 34669, INPA-H 34670, INPA-H 34671, INPA-H 34672, INPA-H 34673, INPA-H 34674, INPA-H 34675, INPA-H 34676, INPA-H 34677, INPA-H 34678).

***Scinax* sp. 3:** BRAZIL: *AMAZONAS*: Berurí, BR-319, km 350 (INPA-H 20582, INPA-H 20586, INPA-H 34585, INPA-H 34584, INPA-H 34581, INPA-H 34583, INPA-H 34587); RONDÔNIA: Porto Velho, UHE Santo Antônio (INPA-H 34591, INPA-H 34590, INPA-H 34589, INPA-H 34592, INPA-H 34595, INPA-H 34588, INPA-H 34594, INPA-H 34593).

***Scinax* sp. 4**: BRAZIL: *AMAZONAS*: Humaitá, BR-319, km 620 (INPA-H 34693).

***Scinax* sp. 5**: BRAZIL: *AMAZONAS*: Tapauá, BR-319, km 450 (INPA-H 34648, INPA-H 34656, INPA-H 34639, INPA-H 34640, INPA-H 34632); Berurí, BR-319, Km 260 (INPA-H 34703, INPA-H 34693, INPA-H 34696); Borba, BR-319, Km 220 (INPA-H 34710).

***Scinax* sp. 6**: BRAZIL: *AMAZONAS*: Careiro da Várzea, BR-319, km 34, Ramal do Purupuru (INPA-H 34597); *RONDÔNIA*: Porto Velho, UHE Santo Antônio (INPA-H 35559, INPA-H 35561, INPA-H 35562, INPA-H 35563, INPA-H 35564, INPA-H 35565, INPA-H 35566, INPA-H 35567, INPA-H 35568).

***Scinax* sp. 7**: BRAZIL: *AMAZONAS*: Careiro da Várzea, BR-319, km 100 (INPA-H 34600, INPA-H 34601, INPA-H 34604, INPA-H 34614, INPA-H 34615, INPA-H 34622, INPA-H 34598, INPA-H 34624, INPA-H 34627, INPA-H 34629), km 168 (INPA-H 34602); Borba, BR-319, km 220 (INPA-H 34610, INPA-H 34620); Berurí, BR-319, km 260 (INPA-H 34608), km 350 (INPA-H 34599, INPA-H 34607, INPA-H 34609, INPA-H 34611, INPA-H 34612, INPA-H 34617, INPA-H 34618, INPA-H 34621, INPA-H 34625, INPA-H 34626, INPA-H 34628, INPA-H 34630); Manicoré, BR-319, km 400 **(**INPA-H 34603, INPA-H 34606, INPA-H 34616, INPA-H 34623); Tapauá, BR-319, km 450, Nascentes do Lago Jari National Park (INPA-H 34665, INPA-H 34613, INPA-H 34619, INPA-H 34605).

Scinax boesemani: SURINAME: *PARAMARIBO* (*SURINAME*): near Zanderij (RMNH12601, holótipo). BRAZIL: *RORAIMA*: Caracaraí, Parque Nacional do Viruá (INPA-H 25972, INPA-H 25974).

Scinax chiquitanus: BRAZIL: *RONDÔNIA*: Porto Velho, UHE Santo Antônio, M-14 (INPA-H 35554, INPA-H 35555, INPA-H 35556, INPA-H 35557, INPA-H 35558, INPA-H 35560).

Scinax cruentommus: ECUADOR: *NAPO*: Santa Cecilia (KU 126587, holótipo); *ORELLANA*: Parque Nacional Yasuní (QCAZ 8184), Río Napo (QCAZ 43772, QCAZ 44754). BRAZIL: *AMAZONAS*: Careiro da Várzea, BR-319, km 34, Ramal do Purupuru (INPA-H 34697).

Scinax* aff. *cruentommus: BRAZIL: *AMAZONAS*: Manicoré, BR-319, Km 300 (INPA-H 34596).

***Scinax funereus*:** ECUADOR: *ORELLANA*: Río Napo, Primavera (QCAZ 43799), Tambococha (QCAZ 55280, QCAZ 55283).

Scinax fuscomarginatus: BRAZIL: *RORAIMA*: Boa Vista, Estação Ecológica de Maracá (INPA-H 34662, INPA-H 34634, INPA-H 34646, INPA-H 34661); Caracaraí, Parque Nacional do Viruá (INPA-H 19371, INPA-H 19372, INPA-H 19376, INPA-H 19378, INPA-H 19383, INPA-H 19384).

Scinax garbei: BRAZIL: *RORAIMA*: Caracaraí, Parque Nacional do Viruá (INPA-H 25964, INPA-H 27496).

Scinax madeirae: BRAZIL: *RONDÔNIA*: Alta Floresta, Parque Estadual Corumbiaria (INPA-H 7050, INPA-H 7051).

Scinax nebulosus: BRAZIL: *PARÁ*: Alter do Chão (INPA-H 34647, INPA-H 34653); *RONDÔNIA*: Costa Marques, Real Forte Príncipe da Beira (INPA-H 34641); *RORAIMA*: Caracaraí, Parque Nacional do Viruá (INPA-H 27535, INPA-H 27536, INPA-H 27537).

Scinax proboscideus: BRAZIL: *AMAZONAS*: Manaus, Reserva Colosso do PDBFF (INPA-H 10304); Presidente Figueiredo, Vila Pitinga (INPA-H 1870); *PARÁ*: Oriximiná, UHE Cachoeira Porteira, Rio Trombetas (INPA-H 304).

***Scinax ruber* F**: BRAZIL: *AMAZONAS*: Borba, BR-319, km 220 (INPA-H 34642, INPA-H 34652); *RONDÔNIA*: Porto Velho, UHE Santo Antônio, (INPA-H 34655, INPA-H 34649, INPA-H 34633, INPA-H 34635).

***Scinax ruber* PM**: BRAZIL: *AMAZONAS*: Careiro da Várzea, AM-354, km 10 (INPA-H 34658, INPA-H 34654, INPA-H 34645, INPA-H 34659).

***Scinax ruber* 1**: COLOMBIA: *SANTANDER*, Sabana de Torres (ANDES-A 1810).

***Scinax ruber* 3**: COLOMBIA: *META*, San Juan de Arama (ANDES-A 1290, ANDES-A 1812); *CASANARE*, Sabanalarga (ANDES-A 1040, ANDES-A 1046; ANDES-A 1504)

Scinax sateremawe: BRAZIL: *AMAZONAS*: Borba, Ramal Novo Horizonte (INPA-H 34695, INPA-H 34708).

***Scinax wandae* A**: COLOMBIA: *META*, San Juan de Arama (ANDES-A 1287, ANDES-A 1814, ANDES-A 1815).

***Scinax wandae* B**: COLOMBIA: *CASANARE*, Sabanalarga (ANDES-A 1234, ANDES-A 1077, ANDES-A 1072).
