# Supplementary material for: High Species Richness of Scinax Treefrogs (Hylidae) in a Threatened Amazonian Landscape Revealed by an Integrative Approach
Source: PLoS One. 2016 Nov 2;11(11):e0165679. doi: 10.1371/journal.pone.0165679 (PMC5091857; doi:10.1371/journal.pone.0165679)
Supplement: S1 Table — (PDF) [file pone.0165679.s003.pdf]

Voucher	Species	Location	GenBank	Authors
MLPA2137	*O. berthae*	Argentina, Buenos Aires, Atalaya	AY843754	Faivovich *et al*. 2005
CFBH5788	*J. uruguayus*	Brazil, Rio Grande do Sul, Cambará do Sul	AY843681	Brusquetti *et al.* 2014
	S. boesemani A	French Guiana, Grand santi	EF217497	Fouquet *et al*. 2007b
	S. boesemani A	French Guiana, Savane roche virginie	EF217498	Fouquet *et al*. 2007b
147mc	*S. boesemani* A	French Guiana, Guatemala	EF217501	Fouquet *et al*. 2007b
	S. boesemani A	French Guiana, Savane roche virginie	EF217500	Fouquet *et al*. 2007b
	S. boesemani B	Suriname, Road to Apura	EU201090	Fouquet *et al*. 2007a
	S. boesemani B	French Guiana, Grand santi	EF217502	Fouquet *et al*. 2007b
	S. boesemani C	Suriname, Sipilawini	EU201089	Fouquet *et al*. 2007a
198mc	*S. boesemani* D	French Guiana, Grand santi	EF217503	Fouquet *et al*. 2007b
39mc	*S. boesemani* D	French Guiana, Grand santi	EF376072	Salducci *et al*. 2005
	S. boesemani E	Guyana, Mazaruni, Potaro Imbaimadai	EU201088	Fouquet *et al*. 2007a
MNKA 9481	*S. chiquitanus* BOL	Bolivia, Santa Cruz, Sara, Buenavista	JF789945	Jansen *et al*. 2011
INPAH35554	*S. chiquitanus* BRA	Brazil, Rondônia, Porto Velho, module 14	KU317380	New
INPAH35555	*S. chiquitanus* BRA	Brazil, Rondônia, Porto Velho, module 14	KU317379	New
INPAH35556	*S. chiquitanus* BRA	Brazil, Rondônia, Porto Velho, module 14	KU317381	New
INPAH35557	*S. chiquitanus* BRA	Brazil, Rondônia, Porto Velho, module 14	KU317382	New
INPAH35558	*S. chiquitanus* BRA	Brazil, Rondônia, Porto Velho, module 14	KU317383	New
INPAH35560	*S. chiquitanus* BRA	Brazil, Rondônia, Porto Velho, module 14	KU317384	New
INPAH34697	*S. cruentommus* BRA	Brazil, Amazonas, BR-319, module 1	KU317385	New
324mc	*S. cruentommus* GUF B	French Guiana, Antecum,Pata	EU201093	Fouquet *et al*. 2007a
	S. cruentommus GUF A1	French Guiana, Kaw	EF217504	Fouquet *et al*. 2007b
	S. cruentommus GUF A2	French Guiana	EF217506	Fouquet *et al*. 2007b
	S. cruentommus GUF A2	French Guiana, Kaw	EF217507	Fouquet *et al*. 2007b
PG 67	*S. cruentommus* GUF A2	French Guiana, Patawa	EF217508	Fouquet *et al*. 2007b
	S. cruentommus GUF A2	French Guiana, Kaw	EF376074	Salducci *et al*. unpublished
	S. cruentommus GUF A2	French Guiana	EF376075	Salducci *et al*. unpublished
8mc	*S. cruentommus* GUF A2	French Guiana, Mountain of Kaw	AF467263	Salducci *et al*. 2002
INPAH34596	*S.* aff. *cruentommus* BRA	Brazil, Amazonas, BR-319, módulo 6	KU317386	New
CFBH24360	*S. fuscomarginatus*	Brazil, Minas Gerais, Lagoa Santa	KJ004134	Brusquetti *et al.* 2014
CFBH24361	*S. fuscomarginatus*	Brazil, Minas Gerais, Lagoa Santa	KJ004135	Brusquetti *et al.* 2014
CFBH24362	*S. fuscomarginatus*	Brazil, Minas Gerais, Lagoa Santa	KJ004136	Brusquetti *et al.* 2014

MNKA 9695	*S. fuscovarius*	Bolivia, Santa Cruz, Ñuflo de Chavez, San Sebastián	JF790013	Jansen *et al.* 2011
MNKA 9772	*S. fuscovarius*	Bolivia, Santa Cruz, Ñuflo de Chavez, San Sebastián	JF790014	Jansen *et al.* 2011
KU 202764	*S. garbei*	Ecuador, Chimborazo	AY326033	Darst & Cannatella 2004
NMP6V 71267-1	*S. iquitorum*	Peru, Puerto Almendras	KU317397	New
NMP6V 71267-3	*S. iquitorum*	Peru, Puerto Almendras	KU317398	New
	S. jolyi	French Guiana	AF467261	Salducci *et al*. 2002
MNKA9353	*S. madeirae*	Bolivia, Beni, Yucuma, Los Lagos	KJ004100	Brusquetti *et al.* 2014
CFBH25469	*S. madeirae*	Brazil, Rondônia, Porto Velho	KJ004101	Brusquetti *et al.* 2014
CFBHT10951	*S. nebulosus*	Brazil, Piauí, Baixa Grande	KJ004190	Brusquetti *et al.* 2014
	S. nebulosus	French Guiana, Road 8/pk6	EF217514	Fouquet *et al*. 2007b
	S. proboscideus	French Guiana, Kaw	EF376070	Salducci *et al*. unpublished
	S. ruber A	French Guiana, Kaw	EF217473	Fouquet *et al*. 2007b
177mc	*S. ruber* A	French Guiana, Ouanary	EF217474	Fouquet *et al*. 2007b
	S. ruber A	French Guiana	EF217475	Fouquet *et al*. 2007b
137bm	*S. ruber* A	French Guiana, Cacao	EF217476	Fouquet *et al*. 2007b
	S. ruber B	French Guiana	EF217481	Fouquet *et al*. 2007b
141bm	*S. ruber* B	French Guiana, Petit saut	EF217482	Fouquet *et al*. 2007b
148bm	*S. ruber* B	French Guiana, Road CSG Sinnamary	EF217483	Fouquet *et al*. 2007b
IWK 109	*S. ruber* C	Guyana, Iwokrama, Muri Scrub camp	AY549365	Faivovich *et al*. 2004
164AF	*S. ruber* C	Suriname, Brownsberg	EU201092	Fouquet *et al*. 2007a
40C	*S. ruber* C	French Guiana	EF376073	Salducci *et al*. 2005
QCAZ25275	*S. ruber* D	Ecuador, parroquia Dayuma,canton coca,Orellana	EF217487	Fouquet *et al*. 2007b
	S. ruber E	French Guiana, Kourou	EF217488	Fouquet *et al*. 2007b
	S. ruber E	French Guiana, Ile royale	EF217489	Fouquet *et al*. 2007b
76mc	*S. ruber* E	French Guiana, Mont Ravel	EF217490	Fouquet *et al*. 2007b
MNKA 9538	*S. ruber* F	Bolivia, Santa Cruz, Velasco, Caparu	JF790033	Jansen *et al*. 2011
MNKA 9539	*S. ruber* F	Bolivia, Santa Cruz, Velasco, Caparu	JF790034	Jansen *et al*. 2011
INPAH34633	*S. ruber* F	Brazil, Rondônia, Porto Velho, módulo 17	KU317402	New
INPAH34701	*S. ruber* F	Brazil, Rondônia, Porto Velho, módulo 16	KU317401	New
INPAH34699	*S. ruber* F	Brazil, Amazonas, BR-319, módulo 4	KU317399	New
INPAH34642	*S. ruber* F	Brazil, Amazonas, BR-319, módulo 4	KU317400	New
INPAH34645	*S. ruber* PM	Brazil, Amazonas, BR-319, módulo 2	KU317404	New
INPAH34652	*S. ruber* PM	Brazil, Amazonas, BR-319, módulo 2	KU317403	New

AJC 2324	*S. ruber* 1	Colombia, Orocué, Casanare	KP149491	Guarnizo *et al*. 2015
AJC 3884	*S. ruber* 1	Colombia, Sabana de Torres, Santander	KP149330	Guarnizo *et al*. 2015
AJC 3532	*S. ruber* 2	Colombia, San Vicente, Santander	KP149347	Guarnizo *et al*. 2015
AJC 3534	*S. ruber* 2	Colombia, San Vicente, Santander	KP149295	Guarnizo *et al*. 2015
AJC 3446	*S. ruber 3*	Colombia, San Juan de Arama, Meta	KP149466	Guarnizo *et al*. 2015
AJC 3378	*S. ruber 3*	Colombia, Sabanalarga, Casanare	KP149452	Guarnizo *et al*. 2015
KU 207622	*S. ruber* Peru	Peru, Madre de Dios, Cusco Amazonico	AY326034	Darst & cannatella 2004
CFBH21975	*S. squalirostris*	Brazil, São Paulo, Serra da Bocaina	KJ004187	Brusquetti *et al.* 2014
CHUNB34502	*S. villasboasi*	Brazil, Pará, Serra do Cachimbo	KJ004103	Brusquetti *et al.* 2014
CHUNB34503	*S. villasboasi*	Brazil, Pará, Serra do Cachimbo	KJ004104	Brusquetti *et al.* 2014
CHUNB40161	*S. villasboasi*	Brazil, Pará, Serra do Cachimbo	KJ004109	Brusquetti *et al.* 2014
AJC 4105	*S. wandae* A	Colombia, Sabanalarga, Casanare	KP149381	Guarnizo *et al*. 2015
AJC 3974	*S. wandae* A	Colombia, Sabanalarga, Casanare	KP149323	Guarnizo *et al*. 2015
AJC 4120	*S. wandae* A	Colombia, Sabanalarga, Casanare	KP149319	Guarnizo *et al*. 2015
AJC 3461	*S. wandae* B	Colombia, San Juan de Arama, Meta	KP149431	Guarnizo *et al*. 2015
AJC 3464	*S. wandae* B	Colombia, San Juan de Arama, Meta	KP149460	Guarnizo *et al*. 2015
AJC 1743	*S. wandae* B	Colombia, San Juan de Arama, Meta	KP149376	Guarnizo *et al*. 2015
144bm	*S. x-signatus*	French Guiana	EF217479	Fouquet *et al*. 2007b
260mc	*S. x-signatus*	French Guiana, Arataï	EF217480	Fouquet *et al*. 2007b
INPAH34688	*Scinax* sp. 1	Brazil, Amazonas, BR-319, module 9	KU317428	New
INPAH34690	*Scinax* sp. 1	Brazil, Amazonas, BR-319, module 9	KU317431	New
INPAH34700	*Scinax* sp. 1	Brazil, Amazonas, BR-319, module 9	KU317430	New
INPAH34670	*Scinax* sp. 2	Brazil, Amazonas, BR-319, module 11	KU317412	New
INPAH34672	*Scinax* sp. 2	Brazil, Amazonas, BR-319, module 11	KU317410	New
INPAH34676	*Scinax* sp. 2	Brazil, Amazonas, BR-319, module 11	KU317414	New
INPAH34671	*Scinax* sp. 2	Brazil, Amazonas, BR-319, module 11	KU317411	New
APL20791	*Scinax* sp. 2	Brazil, Amazonas, BR-319, module 11	KU317413	New
INPAH34586	*Scinax* sp. 3	Brazil, Amazonas, BR-319, module 7	KU317425	New
INPAH34581	*Scinax* sp. 3	Brazil, Amazonas, BR-319, module 7	KU317416	New
INPAH34583	*Scinax* sp. 3	Brazil, Amazonas, BR-319, module 7	KU317422	New
INPAH34585	*Scinax* sp. 3	Brazil, Amazonas, BR-319, module 7	KU317415	New
INPAH35414	*Scinax* sp. 3	Brazil, Amazonas, BR-319, module 7	KU317426	New
INPAH35411	*Scinax* sp. 3	Brazil, Amazonas, BR-319, module 7	KU317421	New

INPAH35413	*Scinax* sp. 3	Brazil, Amazonas, BR-319, module 7	KU317423	New
INPAH34595	*Scinax* sp. 3	Brazil, Rondônia, Porto Velho, module 13	KU317419	New
INPAH34592	*Scinax* sp. 3	Brazil, Rondônia, Porto Velho, module 13	KU317420	New
INPAH34593	*Scinax* sp. 3	Brazil, Rondônia, Porto Velho, module 13	KU317427	New
INPAH34594	*Scinax* sp. 3	Brazil, Rondônia, Porto Velho, module 13	KU317418	New
INPAH34589	*Scinax* sp. 3	Brazil, Rondônia, Porto Velho, module 13	KU317424	New
INPAH34588	*Scinax* sp. 3	Brazil, Rondônia, Porto Velho, module 13	KU317417	New
INPAH34693	*Scinax* sp. 4	Brazil, Amazonas, BR-319, module 11	KU317429	New
INPAH34696	*Scinax* sp. 5	Brazil, Amazonas, BR-319, module 5	KU317378	New
INPAH34703	*Scinax* sp. 5	Brazil, Amazonas, BR-319, module 5	KU317377	New
INPAH34597	*Scinax* sp. 6	Brazil, Amazonas, BR-319, module 1	KU317391	New
INPAH35562	*Scinax* sp. 6	Brazil, Rondônia, Porto Velho, module 17	KU317387	New
INPAH35561	*Scinax* sp. 6	Brazil, Rondônia, Porto Velho, module 18	KU317392	New
INPAH35559	*Scinax* sp. 6	Brazil, Rondônia, Porto Velho, module 14	KU317395	New
INPAH35563	*Scinax* sp. 6	Brazil, Rondônia, Porto Velho, module 13	KU317393	New
INPAH35564	*Scinax* sp. 6	Brazil, Rondônia, Porto Velho, module 17	KU317389	New
INPAH35565	*Scinax* sp. 6	Brazil, Rondônia, Porto Velho, module 17	KU317390	New
INPAH35566	*Scinax* sp. 6	Brazil, Rondônia, Porto Velho, module 17	KU317388	New
INPAH35568	*Scinax* sp. 6	Brazil, Rondônia, Porto Velho, module 13	KU317394	New
INPAH35567	*Scinax* sp. 6	Brazil, Rondônia, Porto Velho, module 18	KU317396	New
INPAH35410	*Scinax* sp. 7	Brazil, Amazonas, BR-319, module 7	KU317405	New
INPAH34610	*Scinax* sp. 7	Brazil, Amazonas, BR-319, module 8	KU317407	New
INPAH34623	*Scinax* sp. 7	Brazil, Amazonas, BR-319, module 8	KU317409	New
INPAH34625	*Scinax* sp. 7	Brazil, Amazonas, BR-319, module 7	KU317406	New
INPAH34705	*Scinax* sp. 7	Brazil, Amazonas, BR-319, module 9	KU317408	New
MNKA 9134	*Scinax* sp. A	Bolivia, Santa Cruz, Ñuflo de Chavez, San Sebastián	JF790036	Jansen *et al*. 2011
MNKA 9428	*Scinax* sp. A	Bolivia, Santa Cruz, Ñuflo de Chavez, San Sebastián	JF790037	Jansen *et al*. 2011

Abbreviations. - **MLPA**, Museo de La Plata, La Plata, Argentina. - **MC,** Christian Marty field numbers. - **MNKA**, Museo de Historia Natural Noel Kempff Mercado, Santa Cruz de la Sierra, Bolivia. - **INPAH**, Instituto Nacional de Pesquisas da Amazônia, Manaus, Brazil. - **PG**, Philippe Gaucher field numbers. - **CFBH**, Collection Célio F.B. Haddad, Universidade Estadual Paulista, Rio Claro, São Paulo, Brazil. - **KU**, Kansas University, Museum of Natural History, Lawrence, EUA. - **NMP6V**, Department of Zoology, National Museum, Prague, Czech Republic. - **BM**, Michel Blanc field numbers. - **IWK**, Field numbers used by Maureen A. Donnelly. - **QCAZ**, Museo de Zoologia de la Pontificia Universidad Catolica del Ecuador, Quito, Ecuador. - **AJC**, Andrew J. Crawford field numbers. - **CHUNB**, Coleção Herpetológica da Universidade de Brasília, Brasília, D.F., Brazil. - **APL**, Albertina P. Lima field numbers. - **AF**, Antoine Fouquet field numbers.
