# Supplementary material for: High Species Richness of Scinax Treefrogs (Hylidae) in a Threatened Amazonian Landscape Revealed by an Integrative Approach
Source: PLoS One. 2016 Nov 2;11(11):e0165679. doi: 10.1371/journal.pone.0165679 (PMC5091857; doi:10.1371/journal.pone.0165679)
Supplement: S3 Table — (PDF) [file pone.0165679.s005.pdf]

	Scinax sp. 1		*Scinax* sp. 2		*Scinax* sp. 3		*Scinax* sp. 4	*Scinax* sp. 5	*Scinax* sp. 6	
	Male (n = 5)	Female (n = 1)	Male (n = 15)	Female (n = 2)	Male (n = 12)	Female (n = 2)	Male (n = 1)	Male (n = 10)	Male (n = 6)	Female (n = 3)
**SVL**	21.50 ± 1.03 (20.20–22.50)	26.45	19.29 ± 0.76 (18.11–20.37)	19.98 ± 1.34 (19.02–20.93)	32.80 ± 1.20 (31.31–34.51)	37.23 ± 2.40 (35.53–38.93)	23.18	31.67 ± 1.41 (29.58–33.87)	25.66 ± 0.57 (25.09–26.66)	27.08 ± 1.76 (25.45–28.94)
**HL**	8.14 ± 0.57 (7.57–8.96)	9.67	6.68 ± 0.32 (6.14–7.21)	7.23 ± 0.31 (7.01–7.45)	12.22 ± 0.32 (11.80–12.67)	13.40 ± 0.44 (13.09–13.71)	8.28	12.24 ± 0.58 (11.40–13.42)	8.61 ± 0.19 (8.45–8.92)	9.23 ± 0.53 (8.91–9.84)
**HW**	7.15 ± 0.29 (6.82–7.59)	8.95	5.84 ± 0.30 (5.50–6.33)	6.04 ± 0.25 (5.86–6.21)	11.52 ± 0.47 (10.91–12.44)	12.60 ± 0.76 (12.06–13.14)	7.61	11.55 ± 0.54 (10.80–12.77)	8.35 ± 0.26 (8.00–8.74)	8.82 ± 0.66 (8.22–9.53)
**ED**	2.70 ± 0.17 (2.50–2.87)	2.99	1.98 ± 0.11 (1.83–2.20)	2.13 ± 0.08 (2.07–2.18)	3.66 ± 0.25 (3.28–4.20)	3.74 ± 0.36 (3.48–3.99)	2.7	3.41 ± 0.17 (3.21–3.68)	2.76 ± 0.13 (2.61–2.93)	2.79 ± 0.25 (2.51–3.00)
**TD**	0.99 ± 0.25 (0.70–1.37)	1.47	0.79 ± 0.09 (0.61–0.94)	0.88 ± 0.16 (0.76–0.99)	2.12 ± 0.16 (1.86–2.37)	2.42 ± 0.04 (2.39–2.44)	1.14	2.02 ± 0.17 (1.85–2.33)	1.51 ± 0.12 (1.36–1.71)	1.63 ± 0.15 (1.49–1.78)
**UEW**	2.15 ± 0.30 (1.64–2.38)	2.10	1.40 ± 0.16 (1.11–1.70)	1.50 ± 0.15 (1.39–1.60)	3.14 ± 0.21 (2.71–3.43)	3.05 ± 0.04 (3.02–3.07)	2.16	2.71 ± 0.23 (2.39–3.12)	2.16 ± 0.14 (1.92–2.33)	2.43 ± 0.22 (2.22–2.66)
**IOD**	2.26 ± 0.18 (2.02–2.50)	2.88	1.78 ± 0.11 (1.56–1.94)	1.90 ± 0.20 (1.76–2.04)	3.27 ± 0.21 (3.00–3.74)	3.78 ± 0.15 (3.67–3.88)	2.59	3.83 ± 0.18 (3.45–4.13)	2.16 ± 0.09 (2.08–2.31)	2.36 ± 0.20 (2.18–2.58)
**IND**	1.74 ± 0.15 (1.55–1.91)	1.90	1.55 ± 0.08 (1.41–1.70)	1.60 ± 0.14 (1.50–1.70)	2.73 ± 0.07 (2.60–2.83)	3.01 ± 0.16 (2.90–3.12)	1.86	2.72 ± 0.13 (2.52–2.97)	1.81 ± 0.10 (1.70–1.95)	1.99 ± 0.05 (1.96–2.05)
**TAL**	5.77 ± 0.19 (5.54–6.00)	6.83	5.61 ± 0.31 (5.05–6.04)	5.90 ± 0.47 (5.56–6.23)	9.01 ± 0.30 (8.56–9.55)	10.14 ± 0.48 (9.80–10.48)	6.12	9.53 ± 0.43 (8.80–10.17)	7.29 ± 0.20 (7.05–7.66)	8.06 ± 0.27 (7.82–8.36)
**FL**	8.84 ± 0.45 (8.46–9.52)	10.64	7.19 ± 0.45 (6.50–7.80)	7.47± 0.33 (7.24–7.70)	13.59 ± 0.47 (12.90–14.40)	16.08 ± 0.93 (15.42–16.74)	10.00	13.02 ± 0.72 (12.00–14.05)	9.61 ± 0.36 (9.01–10.08)	10.38 ± 1.30 (9.17–11.75)
**HAL**	5.91 ± 0.14 (5.74–6.07)	7.27	4.35 ± 0.24 (4.10–4.80)	4.70 ± 0.28 (4.50–4.90)	9.32 ± 0.41 (8.89–10.40)	11.42 ± 0.68 (10.94–11.90)	6.1	8.67 ± 0.28 (8.14–9.15)	6.95 ± 0.27 (6.60–7.25)	7.37 ± 0.86 (6.49–8.20)
**3FD**	1.00 ± 0.08 (0.89–1.10)	1.02	0.68 ± 0.08 (0.50–0.80)	0.70 ± 0.14 (0.60–0.80)	1.75 ± 0.13 (1.51–1.93)	2.20 ± 0.02 (2.18–2.21)	1.14	1.51 ± 0.21 (1.00–1.86)	1.21 ± 0.07 (1.15–1.32)	1.30 ± 0.16 (1.17–1.48)
**4TD**	0.97 ± 0.11 (0.86–1.09)	0.90	0.68 ± 0.06 (0.60–0.80)	0.70 ± 0.14 (0.60–0.80)	1.64 ± 0.12 (1.36–1.81)	2.00 ± 0.08 (1.94–2.06)	1.02	1.41 ± 0.14 (1.08–1.62)	1.16 ± 0.06 (1.07–1.24)	1.27 ± 0.14 (1.14–1.41)
**END**	2.42 ± 0.14 (2.23–2.55)	2.99	2.07 ± 0.12 (1.90–2.31)	2.16 ± 0.13 (2.07–2.25)	3.95 ± 0.18 (3.61–4.17)	4.31 ± 0.07 (4.26–4.36)	2.57	4.08 ± 0.38 (3.42–4.65)	2.83 ± 0.10 (2.71–2.94)	3.03 ± 0.14 (2.88–3.15)
**TL**	10.93 ± 0.28 (10.58–11.16)	13.00	9.19 ± 0.52 (8.25–9.90)	9.82 ± 0.54 (9.44–10.20)	16.99 ± 0.45 (16.29–17.70)	19.44 ± 1.02 (18.72–20.16)	12.06	16.23 ± 0.58 (15.32–17.16)	12.13 ± 0.27 (11.71–12.41)	13.25 ± 0.86 (12.45–14.16)
**THL**	10.15 ± 0.26 (9.81–10.44)	11.90	8.41 ± 0.42 (7.71–9.22)	8.44 ± 1.04 (7.70–9.17)	15.87 ± 0.64 (14.73–16.88)	18.47 ± 1.00 (17.76–19.17)	11.21	15.12 ± 0.54 (14.38–16.00)	11.54 ± 0.18 (11.28–11.72)	12.34 ± 0.88 (11.35–13.02)

Abbreviations. - **SVL**, snout-vent length. - **HL**, head length. - **HW**, head width. - **ED**, eye diameter. - **TD**, tympanum diameter. - **UEW**, upper eyelid width. - **IOD**, interorbital distance. - **IND**, internarial distance. - **END**, eye-nostril distance. - **TAL**, tarsus length. - **FL**, foot length. - **HAL**, hand length. - **3FD**, third-finger disk diameter. - **4TD**, four-toe disk diameter. -**TL**, tibia length. - **THL**, thigh length. - **n**, number of specimens.

	Scinax sp. 7		*Scinax chiquitanus*		*Scinax cruentommus*	*Scinax* aff. *cruentommus*	*Scinax ruber* F		*Scinax ruber* PM
	Male (n = 28)	Female (n = 7)	Male (n = 4)	Female (n = 2)	Female (n = 1)	Male (n = 1)	Male (n = 2)	Female (n = 2)	Male (n = 5)
**SVL**	24.39 ± 0.87 (22.64–25.92)	26.31 ± 0.73 (25.40–27.50)	30.60 ± 0.59 (29.80–31.15)	34.22 ± 0.73 (33.70–34.73)	30.36	25.46	36.13 ± 1.11 (35.34–36.91)	38.29 ± 0.05 (38.25–38.33)	32.53 ± 1.76 (29.82–34.21)
**HL**	8.95 ± 0.41 (8.30–9.78)	9.57 ± 0.16 (9.39–9.75)	10.49 ± 0.15 (10.35–10.69)	11.59 ± 0.11 (11.51–11.67)	10.63	9.1	12.05 ± 0.33 (11.82–12.28)	13.50 ± 0.05 (13.46–13.54)	11.42 ± 0.44 (10.74–11.92)
**HW**	8.36 ± 0.28 (7.85–8.90)	9.10 ± 0.40 (8.56–9.61)	9.67 ± 0.22 (9.45–9.88)	10.92 ± 0.09 (10.85–10.98)	10.05	8.42	11.67 ± 0.08 (11.61–11.72)	12.88 ± 0.09 (12.82–12.95)	10.54 ± 0.47 (9.81–11.10)
**ED**	3.00 ± 0.20 (2.68–3.41)	3.00 ± 0.15 (2.85–3.24)	3.09 ± 0.08 (3.02–3.21)	3.24 ± 0.01 (3.23–3.25)	3.47	3.3	3.49 ± 0.21 (3.34–3.63)	3.75 ± 0.17 (3.63–3.87)	3.41 ± 0.16 (3.12–3.54)
**TD**	1.39 ± 0.12 (1.11–1.59)	1.46 ± 0.07 (1.38–1.58)	1.58 ± 0.07 (1.51–1.67)	1.91 ± 0.01 (1.90–1.91)	1.76	1.29	2.10 ± 0.05 (2.06–2.13)	2.22 ± 0.07 (2.17–2.28)	2.00 ± 0.07 (1.89–2.09)
**UEW**	2.33 ± 0.20 (1.79–2.59)	2.41 ± 0.22 (2.10–2.69)	2.39 ± 0.26 (2.15–2.72)	2.69 ± 0.12 (2.60–2.77)	2.85	2.08	2.97 ± 0.21 (2.82–3.12)	2.92 ± 0.17 (2.80–3.05)	2.59 ± 0.21 (2.35–2.81)
**IOD**	2.38 ± 0.12 (2.20–2.69)	2.53 ± 0.15 (2.36–2.78)	2.71 ± 0.15 (2.53–2.84)	3.04 ± 0.44 (2.73–3.35)	2.60	2.02	2.97 ± 0.03 (2.95–2.99)	3.37 ± 0.28 (3.17–3.57)	2.75 ± 0.25 (2.40–3.07)
**IND**	1.81 ± 0.10 (1.53–1.96)	1.90 ± 0.12 (1.75–2.08)	2.19 ± 0.10 (2.06–2.30)	2.46 ± 0.07 (2.41–2.51)	2.17	1.96	2.48 ± 0.11 (2.40–3.55)	2.82 ± 0.01 (2.82–2.83)	2.37 ± 0.12 (2.27–2.57)
**TAL**	6.60 ± 0.23 (6.02–7.20)	7.23 ± 0.34 (6.83–7.87)	9.43 ± 0.43 (9.04–9.85)	10.75 ± 0.35 (10.50–11.00)	8.78	7.3	10.62 ± 0.49 (10.27–10.96)	11.58 ± 0.20 (11.44–11.72)	9.77 ± 0.72 (8.91–10.82)
**FL**	9.55 ± 0.44 (8.6–10.26)	10.32 ± 0.45 (9.54–10.84)	12.21 ± 0.33 (11.96–12.68)	12.21 ± 0.33 (11.96–12.68)	11.03	9.28	14.19 ± 0.09 (14.12–14.25)	15.21 ± 0.23 (15.05–15.38)	12.93 ± 0.71 (11.81–13.62)
**HAL**	6.47 ± 0.35 (5.90–7.62)	7.15 ± 0.53 (6.23–7.79)	8.06 ± 0.28 (7.75–8.40)	8.06 ± 0.28 (7.75–8.40)	7.75	6.68	9.81 ± 0.16 (9.70–9.92)	10.58 ± 0.59 (10.16–11.00)	8.71 ± 0.51 (8.19–9.44)
**3FD**	0.94 ± 0.13 (0.72–1.21)	1.02 ± 0.14 (0.87–1.22)	1.50 ± 0.09 (1.40–1.60)	1.50 ± 0.09 (1.40–1.60)	1.28	1.14	1.85 ± 0.13 (1.75–1.94)	1.84 ± 0.07 (1.79–1.89)	1.60 ± 0.10 (1.50–1.77)
**4TD**	0.96 ± 0.14 (0.72–1.26)	0.96 ± 0.10 (0.83–1.11)	1.45 ± 0.14 (1.26–1.58)	1.45 ± 0.14 (1.26–1.58)	1.26	1.15	1.63 ± 0.13 (1.53–1.72)	1.67 ± 0.19 (1.54–1.81)	1.48 ± 0.11 (1.29–1.55)
**END**	2.81 ± 0.17 (2.44–3.05)	3.08 ± 0.15 (2.90–3.30)	3.32 ± 0.13 (3.17–3.47)	3.32 ± 0.13 (3.17–3.47)	3.02	2.74	3.98 ± 0.03 (3.96–4.00)	4.74 ± 0.20 (4.60–4.88)	3.60 ± 0.58 (2.58–4.02)
**TL**	12.25 ± 0.49 (11.19–13.12)	13.42 ± 0.49 (12.87–14.24)	15.41 ± 0.33 (15.12–15.2)	15.41 ± 0.33 (15.12–15.2)	14.18	11.69	18.37 ± 0.50 (18.01–18.72)	19.75 ± 0.39 (19.47–20.02)	17.38 ± 1.06 (16.55–18.88)
**THL**	11.50 ± 0.48 (10.15–12.12)	12.19 ± 1.01 (10.19–13.40)	15.26 ± 0.30 (13.93–14.51)	15.26 ± 0.30 (13.93–14.51)	13.34	11.68	17.51 ± 0.50 (17.15–17.86)	18.78 ± 0.09 (18.71–18.84)	15.18 ± 1.19 (13.32–16.57)

Abbreviations. - **SVL**, snout-vent length. - **HL**, head length. - **HW**, head width. - **ED**, eye diameter. - **TD**, tympanum diameter. - **UEW**, upper eyelid width. - **IOD**, interorbital distance. - **IND**, internarial distance. - **END**, eye-nostril distance. - **TAL**, tarsus length. - **FL**, foot length. - **HAL**, hand length. - **3FD**, third-finger disk diameter. - **4TD**, four-toe disk diameter. -**TL**, tibia length. - **THL**, thigh length. - **n**, number of specimens.
