# Supplementary material for: High Species Richness of Scinax Treefrogs (Hylidae) in a Threatened Amazonian Landscape Revealed by an Integrative Approach
Source: PLoS One. 2016 Nov 2;11(11):e0165679. doi: 10.1371/journal.pone.0165679 (PMC5091857; doi:10.1371/journal.pone.0165679)
Supplement: S4 Table — (PDF) [file pone.0165679.s006.pdf]

Parameters	23)	***Scinax* sp. 1** (n = 21)	***Scinax* sp. 2** (n = 30)	***Scinax* sp. 3** (n = 15)	***Scinax* sp. 7 (**n = 20)
Note duration (s)	0.269 ± 0.025	0.106 ± 0.005 (0.097–0.115)	0.333 ± 0.028	0.110 ± 0.005	0.20 ± 0.05 (0.134–0.331)
Number of pulses	35.5 ± 3.6 (29–41)	25.1 ± 1.1 (23–27)	79.8 ± 6.8 (69–93)	16.8 ± 0.8 (16–18)	14.5 ± 3.4 (10–23)
Pulse duration (s)	(0.007–0.009)	0.002 ± 0.0005 (0.002–0.003)	(0.004–0.005)	(0.004–0.007)	0.008 ± 0.001 (0.007–0.009)
Pulse rate	130.3 ± 6.7 (120–143)	225 ± 25 (200–250)	243 ± 14.3 (200–250)	155.5 ± 19 (143–200)	66.6 ± 3.6 (59–71)
Dominant frequency (Hz)	(2153.3–2153.3) Hz	(2540.9–3014.6) Hz	(4328.2–5081.8) Hz	Hz	(1808.8–1894.9) Hz

Parameters of the advertisement call of *Scinax* species from the Purus-Madeira interfluve and East bank of the upper Madeira river, Brazilian Amazon. Abbreviations. - **n**, number of analyzed calls. - **s**, seconds. - **Hz**, Hertz.
